# Modeling intercellular communication in tissues using spatial graphs of cells

**DOI:** 10.1038/s41587-022-01467-z

**Published:** 2022-10-27

**Authors:** David S. Fischer, Anna C. Schaar, Fabian J. Theis

**Affiliations:** 1 Institute of Computational Biology, Helmholtz Zentrum München, Neuherberg, Germany; 2 TUM School of Life Sciences Weihenstephan, Technical University of Munich, Freising, Germany; 3 Department of Mathematics, Technical University of Munich, Garching bei München, Germany

**Keywords:** Biotechnology, Sequencing

## Abstract

Models of intercellular communication in tissues are based on molecular profiles of dissociated cells, are limited to receptor–ligand signaling and ignore spatial proximity in situ. We present node-centric expression modeling, a method based on graph neural networks that estimates the effects of niche composition on gene expression in an unbiased manner from spatial molecular profiling data. We recover signatures of molecular processes known to underlie cell communication.

## Abstract (web summary)

How cells in a tissue communicate is modeled with a graph neural network.

## Main

**Fig. 1:**
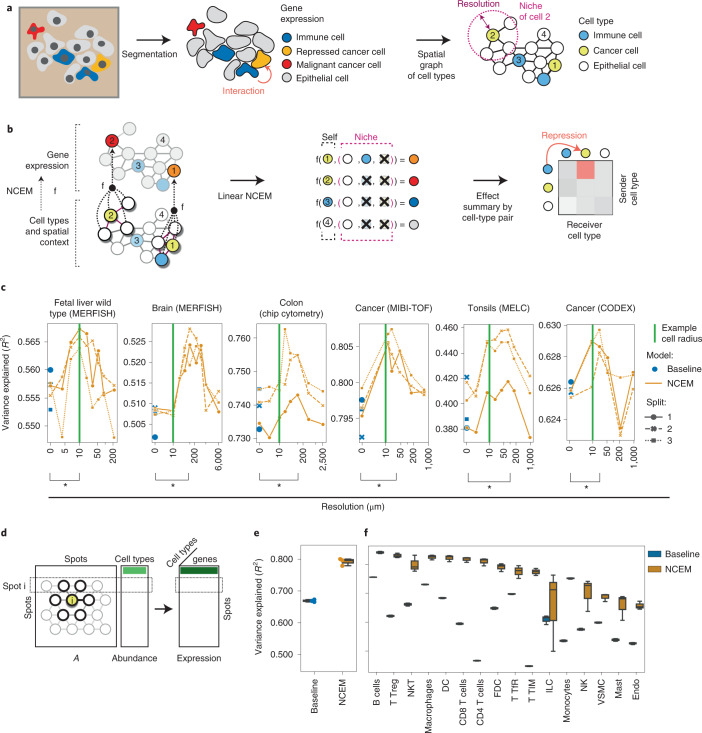
Node-centric expression models capture statistical dependencies between cells in space. **a**, Spatial graphs of cells are based on segmentation masks of cells in spatial molecular profiling data. Resolution is the radius of neighborhood used to define a niche. Numbers label cells and are used in Fig. 1b. **b**, NCEMs describe the gene expression observation of a cell as a function (f) of its spatial neighborhood (niche). **c**, Linear models capture neighborhood dependencies in spatially resolved single-cell data. Shown are the *R*^2^ values between predicted and observed expression vectors on held-out test cells by resolution for six datasets. Green line, 10 µm; baseline (blue points with cross-validation split indicated as shape), a nonspatial linear model; bracket (*), significant difference in paired *t*-test. **d**, Variation in deconvoluted expression vectors over spots for a given cell type can be attributed to spot composition with a linear NCEM. A, spot adjacency matrix. **e**,**f**, NCEM performance on deconvoluted data. Shown are the *R*^2^ values between predicted and observed gene expression vectors for held-out test spots of a linear NCEM in comparison to a baseline model that does not use the spot composition information. The performance is shown across the entire test set (**e**) and split by cell type (**f**) (*n* = 3 cross-validation splits). For each box in (**e**,**f**), the centerline defines the median, the height of the box is given by the interquartile range (IQR), the whiskers are given by 1.5 × IQR and outliers are given as points beyond the minimum or maximum whisker.

**Extended Data Fig. 1:**
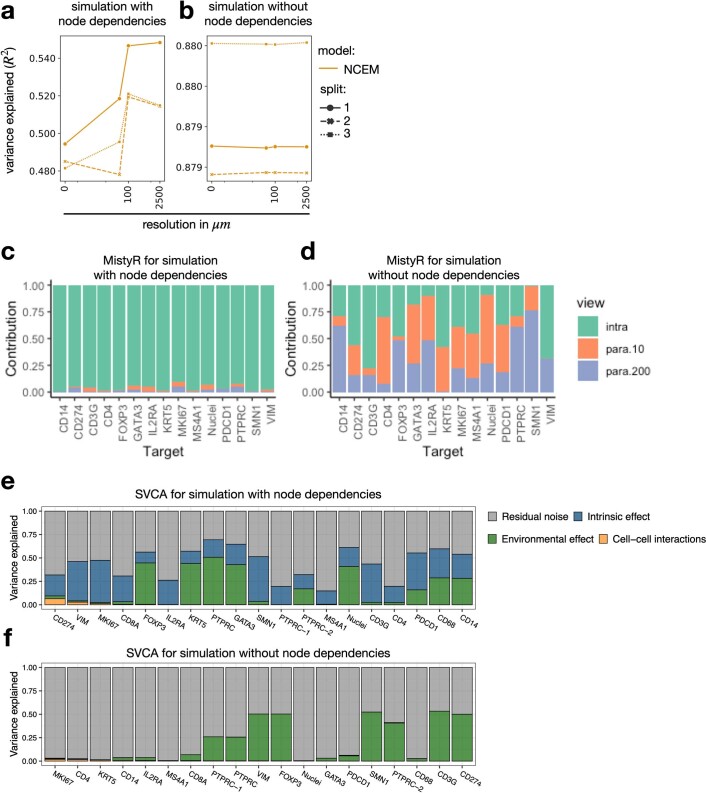
Benchmarking cell–cell dependency inference on simulated data. Shown are fits of NCEMs **(a, b)**, MistyR **(c, d)**, and SVCA **(e, f)** on simulated data with simulated dependencies between cells **(a, c, e)** and without simulated dependencies between cells **(b, d, f)**. The simulation is described in the Methods.

Cells interact on multiple length-scales through direct contact of surface-bound receptors and ligands, tight junctions and mechanical effects, and through indirect mechanisms, including soluble factors. Usually, these communication events cannot be directly observed but are critical to understand emergent phenomena in tissue niches^1^. Molecular signatures of sender and receiver cell types are used to infer latent cell communication events in a tissue through co-occurrence of ligand and receptor expression across putatively communicating cell types^2,3^ and through gene expression signatures in the receiving cell^2,4^. Here we propose node-centric expression models (NCEM) to improve cell communication inference through the use of spatial graphs of cells to constrain axes of cellular communication. We infer cell communication from image-structured molecular profiling assays of RNA or proteins with subcellular resolution (Fig. 1a). We defined an NCEM as a graph neural network that predicts a cell’s observed gene expression vector from its cell type label and its niche^5^ (Fig. 1b and Methods). Cell–cell dependencies may be caused by diverse molecular mechanisms not limited to ligand–receptor-based communication. Therefore, we consider the effects of niche composition on all genes in an unbiased manner. Previous mathematical models of cell–cell interactions in spatial data differed in at least one out of the following central design choices that constitute an NCEM (Supplementary Table 1): they did not represent statistical dependencies of gene expression^6,7^, did not model cell communication events^6–8^, did not work on targeted protocols with limited ligand and receptor gene capture^8–10^ or relied on leave-one-gene hold-outs^4,9^, which can result in false discoveries of dependencies (Extended Data Fig. 1 and Methods).

**Extended Data Fig. 2:**
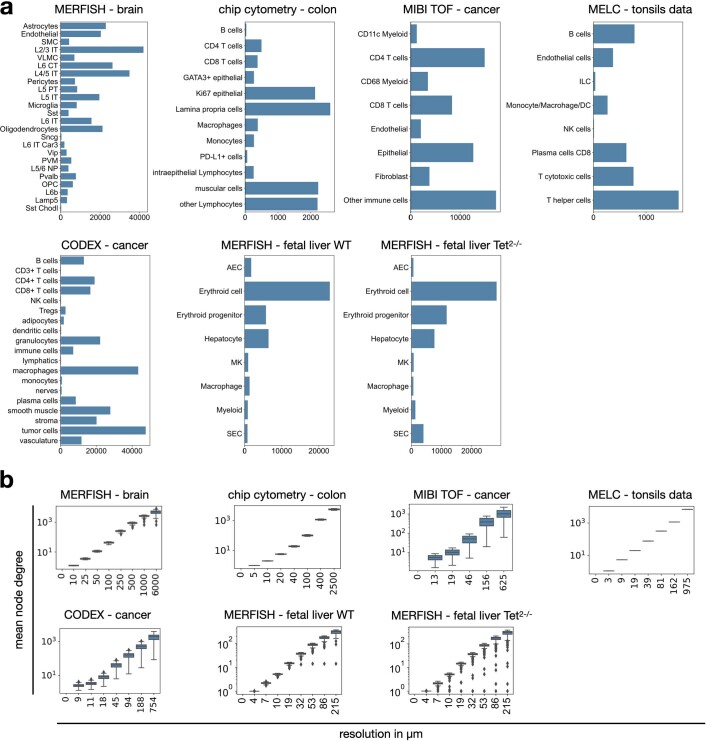
Cell-type centric summary statistics of the considered datasets. **(a)** Cell-type frequencies by dataset. Shown is a barplot with the number of cells in each cell-type for MERFISH – brain data, chip cytometry – colon data, MIBI TOF – cancer data, MELC – tonsils data, CODEX – cancer data, MERFISH - fetal liver wild type and MERFISH - fetal liver *Tet2*−/−. **(b)** Mean node degree (number of neighbors) by resolution in µm and dataset for MERFISH – brain data (n = 284,098 cells), chip cytometry – colon data (n = 11.321 cells), MIBI TOF – cancer data (n = 63,747 cells), MELC – tonsils data (n = 9,512 cells), CODEX – cancer data (n = 272,266 cells), MERFISH - fetal liver wild type (n = 40,864 cells) and MERFISH - fetal liver *Tet2*−/− (n = 54,970 cells). For each box in **(b)**, the centerline defines the median, the height of the box is given by the interquartile range (IQR), the whiskers are given by 1.5 * IQR and outliers are given as points beyond the minimum or maximum whisker.

**Extended Data Fig. 3:**
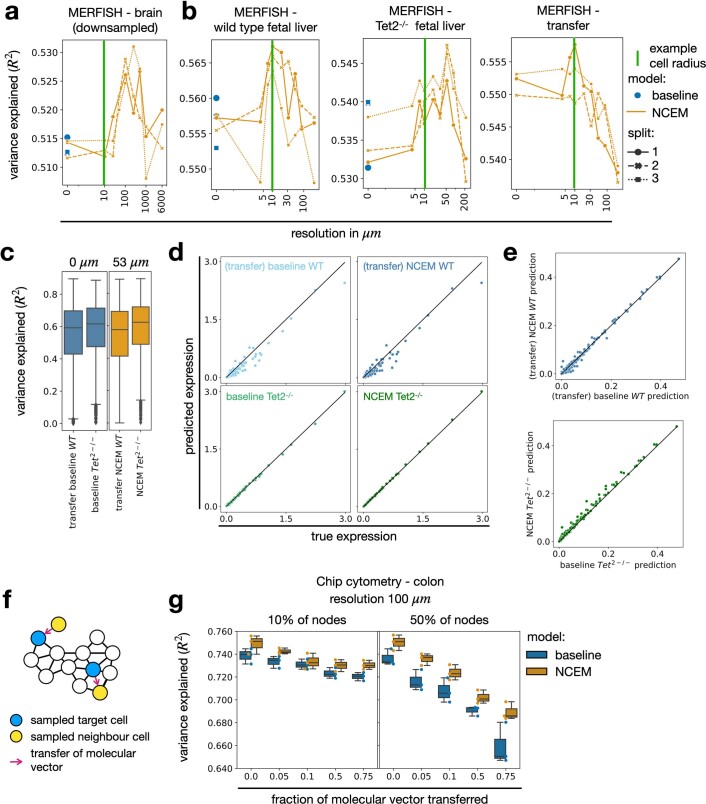
NCEM robustness with respect to data perturbation. **(a)** Robustness of length scales of spatial dependencies to data down-sampling. Shown are the R^2^ values between predicted expression vectors and observed expression vectors for held-out test cells of linear models by resolution in μm with cross validation indicated as point shape and line style, showing the relative performance of NCEM model and baseline model. The downsampling was performed on the full set of images. **(b)** Out-of-domain generalization of NCEM fits across genotypes. We translated a linear NCEM fit on wild type data to *Tet2*-/- knockout data and found a similar spatial dependency structure as in a linear NCEM fit on the knockout data alone. **(c)** Comparison of baseline and optimal resolution linear NCEM fits between the transferred wild type model and the knockout fit on knockout test data, based on R2 values between predicted expression vectors and observed expression vectors (n = 4,740 cells). **(d)** Comparison of true and predicted gene-wise mean expression for different models on knockout evaluation data. **(e)** Comparison of R2 values attained by NCEM and baseline model in transfer and in within-domain prediction task. **(f)** Concept of simulation of segmentation errors. Cells from a measured spatial graph are sampled at random and a fraction of their molecular counts is transferred to neighboring cells, simulating a misplacement of a segmentation boundary between both cells (Methods). **(g)** Robustness in terms of segmentation errors for baseline and NCEM linear model on the chip cytometry - colon dataset for 10% and 50% of all nodes in the dataset and different strengths of augmentation (n = 3 cross-validation splits). For each box, the centerline defines the median, the height of the box is given by the interquartile range (IQR), the whiskers are given by 1.5 * IQR and outliers are given as points beyond the minimum or maximum whisker.

We demonstrate cell communication inference with NCEMs on six datasets measured with MERFISH^11,12^, CODEX^13^, MIBI-TOF^14^, MELC^15^ and chip cytometry^16^ (Extended Data Fig. 2 and Methods). On average, intracell-type variance accounted for 40.6% of the total variance (Supplementary Fig. 1 and Methods). We defined the screened neighborhood sizes such that they cover the range of average node degrees of the given dataset (Extended Data Fig. 2b). We fit a linear model of gene expression based on a niche represented as interaction terms between the receiver cell type and the presence of each (sender) cell type in the neighborhood (Methods). Linear NCEMs were most predictive on an intermediate length scale of 69 µm across the six datasets (Fig. 1c), showing that cell–cell dependencies appear on length scales characteristic of molecular mechanisms of cell communication. NCEMs outperformed nonspatial baseline models consistently by an average Δ*R*^2^ (Online Methods) of 0.016 (Fig. 1c). As expected, the Δ*R*^2^ is small compared to the baseline model *R*^2^ that characterizes between-cell-type variance (0.39–0.79) because cell type identity accounts for a large fraction of variance in single-cell gene expression assays^17^. The inferred length scales were robust to data downsampling (Extended Data Fig. 3a), out-of-domain data from an unseen genetic knockout condition (Extended Data Fig. 3b–e), to simulated segmentation errors (Extended Data Fig. 3f,g) and to removal of the interaction terms from the linear model (Supplementary Fig. 2). The spatial effect on model performance varies between target cell types, suggesting that cell-type-specific molecular mediators of cell–cell dependency are captured (Supplementary Fig. 3).

**Extended Data Fig. 4:**
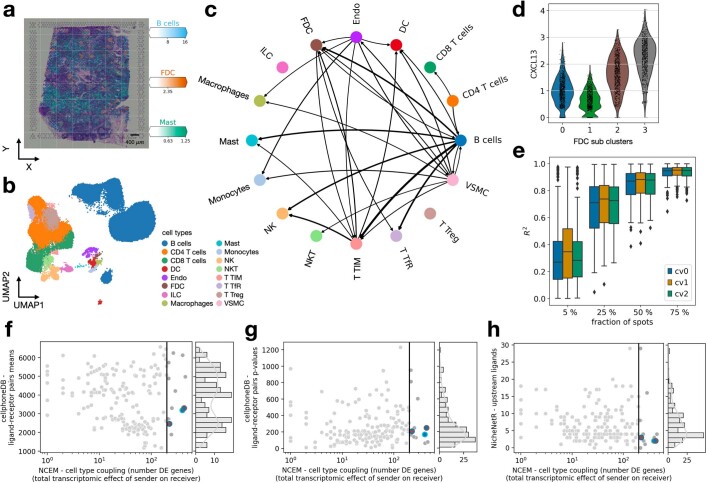
Robustness of cell communication inference on deconvoluted spot transcriptomics data. **(a)** 10x Visium slide of a lymph node with the spot-wise abundance of B cells, follicular dendritic cells (FDC), and mast cells inferred with cell2location superimposed. **(b)** UMAP of cells in a matched scRNA-seq data set of human lymph nodes, spleen and tonsils with cell types superimposed. **(c)** Type coupling heatmap of the Visium – lymph node dataset, with edge width proportional to the number of differentially expressed genes at a false-discovery-rate-corrected p-value threshold of 0.05 for each pair of sender and receiver cell types. Only edges with at least 200 genes are shown. **(d)** Violin plot of *Cxcl13* expression per cell for FDC subclusters in the Visium - lymph node data. **(e)** Robustness of type coupling analysis in a Visium slide on human lymph nodes. Shown are R^2^ between inferred cell type coupling vectors of randomly subsampled spots and the complete data for subsampling ratios of 5 %, 25 %, 50% and 75 % in three cross validations (n = 256 type couplings in each boxplot). For each box, the centerline defines the median, the height of the box is given by the interquartile range (IQR), the whiskers are given by 1.5 * IQR and outliers are given as points beyond the minimum or maximum whisker. **(f-h)** Correlation of measures of cell communication events between pairs of cell types compared with type coupling scores from NCEM on the tabular sapiens lymph node dataset. Shown are CellphoneDB permutation test results with the number of ligand-receptor pairs with positive mean expression **(f)**, number of ligand-receptor pairs with a FDR-corrected p-value below a threshold of 0.05 **(g)** and the number of ligands associated with a pair of cell types as identified by NicheNet **(h)** (Methods). Each point is one pair of cell types. The vertical line indicates the threshold for showing edges in Fig. 2b.

NCEMs can be extended to spot transcriptomics if within-cell-type variation can be recovered from spot transcriptomics datasets in deconvolution analyses^18,19^. Here NCEMs model the expression variation within cell types across spots as a function of the inferred spot composition (Fig. 1d and Methods). We considered data from lymph nodes^18,19^ (Extended Data Fig. 4a,b) for which a deconvolution was previously demonstrated with cell2location^19^. Linear NCEMs were substantially better at predicting gene expression states of cell types in particular spots than a nonspatial baseline model, both globally (Fig. 1e) and for each cell type (Fig. 1f). The inferred couplings were stable to moderate subsampling of the transcriptomics spots in the training data (Extended Data Fig. 4d). We found putatively interacting ligand–receptor pairs for almost all type couplings in CellPhoneDB^3^ and NicheNet^2^ analyses of matched single-cell RNA sequencing (scRNA-seq) data, thus demonstrating the need for a quantitative description of statistical couplings in niches (Extended Data Fig. 4e–g). We also identified spatial dependencies between entire niches when modeling spot graphs (Supplementary Fig. 4).

**Extended Data Fig. 5:**
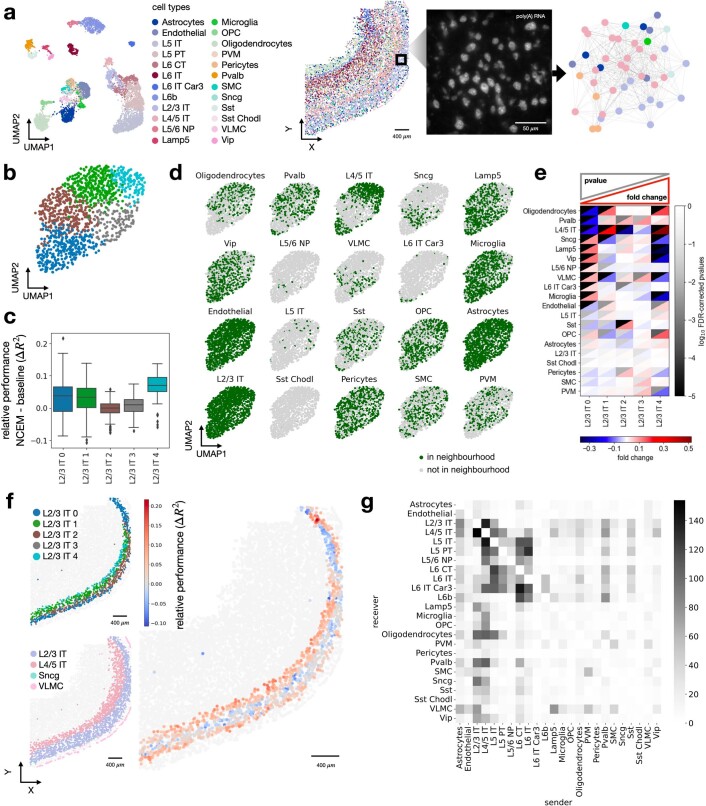
Cell heterogeneity can be attributed to niche composition. **(a)** Spatial cell type distribution in the mouse brain. Shown are a UMAP of molecular embedding of all cells in slide 153 (n = 7439 cells) with the cell type superimposed, followed by slice 153 of mouse brain in the MERFISH – brain dataset with the spatial allocation of all cell types superimposed, field of view number 486 of the same slice with poly(A) RNA channel superimposed at central z-plane (z = 4.5 µm), and the spatial proximity graph of the same field of view with a resolution of 100 µm. **(b)** UMAPs of molecular embedding of L2/3 IT cells with molecular subclustering superimposed (colors as in b)**. (c)** Distribution of cell-wise difference of R^2^ between NCEM and non-spatial baseline model by molecular subcluster (L2/3 IT 0: n = 316, L2/3 IT 1: n = 314, L2/3 IT 2: n = 313, L2/3 IT 3: n = 133, L2/3 IT 4: n = 128). The centerline of the boxplots defines the median, the height of the box is given by the interquartile range (IQR), the whiskers are given by 1.5 * IQR and outliers are given as points beyond the minimum or maximum whisker. **(d)** UMAPs of molecular embedding of all L2/3 IT cells in an example image (n = 1204 cells) showing if a given cell-type is present in the neighborhood. The underlying neighborhoods were defined at the optimal resolution defined in Fig. 1d (100 µm). **(e)** Heatmap of fold change and false-discovery rate corrected p-values of cluster enrichment of binary neighborhood labels, where fold changes are the ratio between the relative neighboring source cell-type frequencies per subtype cluster and the overall source cell-type frequency in the image. **(f)** Model performance on L2/3 IT cells in space on slice 153 of mouse brain in the MERFISH – brain dataset with L2/3 IT sub-states (first panel), L2/3 IT, L4/5 IT, Sncg, and VLMC (second panel) and the difference of R2 between the NCEM at resolution of 100 µm and the best nonspatial baseline model (third panel) superimposed. **(g)** Type coupling analysis of MERFISH – brain data, showing the number of differentially expressed genes at a false-discovery-rate-corrected p-value threshold of 0.05 for each pair of sender and receiver cell types.

**Extended Data Fig. 6:**
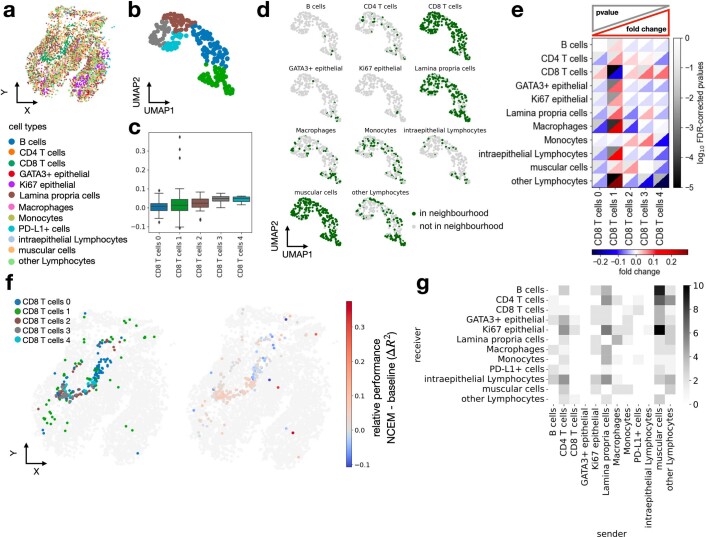
Attributing cell heterogeneity to niche composition in inflamed colon. **(a)** Area 1 of chip cytometry – colon dataset with cell-types superimposed. **(b)** UMAPs of molecular embedding of CD8 T cells only with molecular subclustering superimposed (colors as in c)**. (c)** Distribution of cell-wise difference of R^2^ between spatial model non nonspatial baseline model by molecular sub-cluster (CD8 T cells 0: n = 74, CD8 T cells 1: n = 58, CD8 T cells 2: n = 41, CD8 T cells 3: n = 37, CD8 T cells 4: n = 24). The centerline of the boxplots defines the median, the height of the box is given by the interquartile range (IQR), the whiskers are given by 1.5 * IQR and outliers are given as points beyond the minimum or maximum whisker. **(d)** UMAPs of molecular embedding of all CD8 T cells in area 1 (n = 234 cells) showing if a given cell-type is present in the neighborhood. The underlying neighborhoods were defined at the best performing resolution identified in Fig. 1c (40 µm). **(e)** Heatmap of fold change and false-discovery rate corrected p-values of cluster enrichment of binary neighborhood labels, where fold changes are the ratio between the relative neighboring source cell-type frequencies per subtype cluster and the overall source cell-type frequency in the image. **(f)** Area 1 of colon in the chip cytometry – colon dataset with CD8 T cell sub-states (left) and the difference of R^2^ between the NCEM interaction model at resolution of 40 µm and the best nonspatial baseline model (right). **(g)** Type coupling analysis, showing the number of differentially expressed genes at a false-discovery-rate-corrected p-value threshold of 0.05 for each pair of sender and receiver cell types.

**Extended Data Fig. 7:**
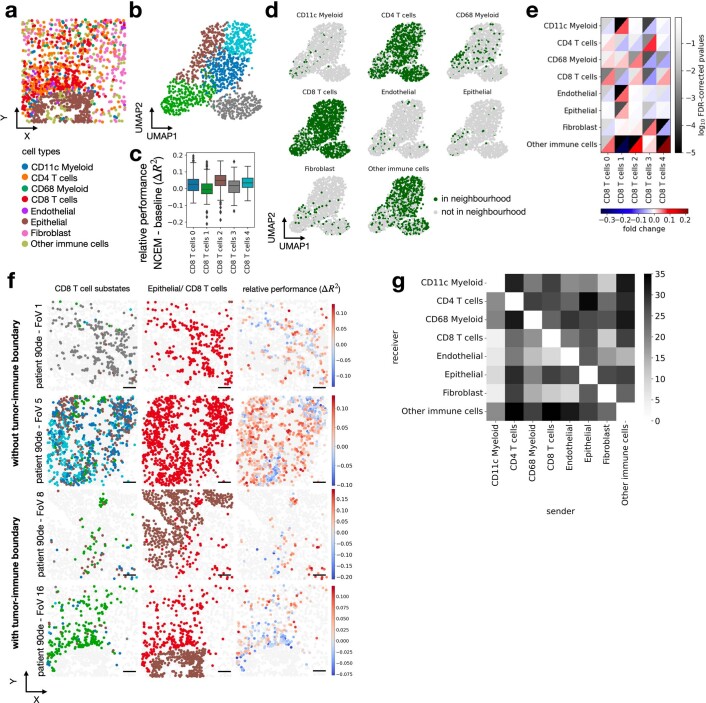
Attributing cell heterogeneity to niche composition in colorectal cancer. **(a)** Field of view 16 of MIBI TOF – cancer dataset with the spatial allocation of all cell-types superimposed. **(b)** UMAPs of molecular embedding of CD8 T cells only with molecular sub-clustering superimposed (colors as in c)**. (c)** Distribution of cell-wise difference of R^2^ between spatial model non-spatial baseline model by molecular sub-cluster (CD8 T cells 0: n = 304, CD8 T cells 1: n = 293, CD8 T cells 2: n = 278, CD8 T cells 3: n = 247, CD8 T cells 4: n = 207). The centerline of the boxplots defines the median, the height of the box is given by the interquartile range (IQR), the whiskers are given by 1.5 * IQR and outliers are given as points beyond the minimum or maximum whisker. **(d)** UMAPs of molecular embedding of all CD8 T cells in area 1 (n = 1,329 cells) showing if a given cell type is present in the neighborhood. The underlying neighborhoods were defined at the optimal resolution identified in Fig. 1d (13 µm). **(e)** Heatmap of fold change and false-discovery rate corrected p-values of cluster enrichment of binary neighborhood labels, where fold changes are the ratio between the relative neighboring source cell-type frequencies per subtype cluster and the overall source cell-type frequency in the image. **(f)** Field of view 1, 5, 8 and 16 of colon in the MIBI TOF – cancer dataset with CD8 T cell sub-states, cell type assignments to epithelial and T cells, and the difference of R^2^ between the NCEM interaction model at a resolution of 13 µm and the best nonspatial baseline model (scale bar 50 µm). **(g)** Type coupling analysis, showing the number of differentially expressed genes at a false-discovery-rate-corrected p-value threshold of 0.05 for each pair of sender and receiver cell types.

Next we interpreted the spatial dependencies in the MERFISH brain data. We found that L2/3 intratelencephalic (IT) cells depend on the presence of Sncg expressing cells, vascular leptomeningeal cells, and L4/5 cells in their niche (Extended Data Fig. 5 and Supplementary Fig. 5). These associations are reproduced by CellPhoneDB (Supplementary Fig. 6a,b). The L2/3 IT cell subclusters are spatially localized in distinct areas of the primary motor cortex^12^. Indeed, the relative performance of NCEM is spatially structured (Extended Data Fig. 5f and Supplementary Fig. 5e). We quantified these dependencies between cell types as ‘cell type couplings’, the number of significant gene-wise coefficients of the cell type pair in an NCEM fit (Extended Data Fig. 5g and Methods). We discovered a dependency of CD8 T cells on multiple other cell types in the chip cytometry colon data (Extended Data Fig. 6) and a dependency of CD8 T cells on proximity to the tumor–immune boundary^14^ in colorectal cancer (Extended Data Fig. 7).

**Fig. 2:**
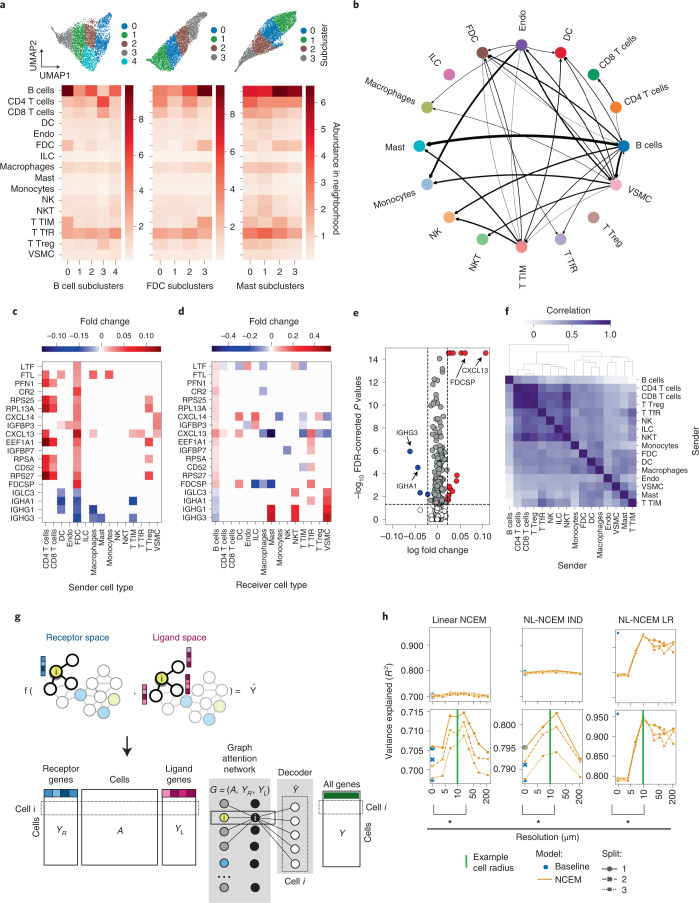
Node-centric expression models identify interacting cell types. **a**, Top: UMAPs with subclusterings of spot-wise gene expression of B cells, FDCs and mast cells from the Visium lymph node dataset. Bottom: the average abundance of cell types per neighborhood after deconvolution of the subclusters. **b**, Type coupling analysis with edge width proportional to the L1 norm of the vector of fold changes of differentially expressed genes for each pair of sender and receiver cell types. Only edges with at least 200 genes are shown. **c**,**d**, Sender effect (**c**) and receiver effect (**d**) analysis of the FDC–B cell signaling axis. Shown is the estimated fold change that the sender cell type on the *x* axis induces in the gene on the *y* axis in receiving B cell **(c)** and conversely for receiver cell types given sender FDCs (**d**). **e**, Volcano plot of differentially expressed genes of B cells in the neighborhood of FDC. **f**, Sender similarity analysis based on a correlation of the coefficient vectors of each sender type with respect to B cell receivers. **g**, A graph kernel to specifically model receptor activity of a cell given the observed ligand expression in its niche extends NCEM to ligand–receptor modeling. **h**, *R*^2^ values between predicted and observed expression vectors for held-out test cells of linear (NCEM), nonlinear (NL-NCEM IND) and ligand–receptor-kernel NCEMs (NL-NCEM LR) by resolution on imputed MERFISH fetal liver data. Green line, 10 µm; baseline (blue points with cross-validation split indicated as shape), a nonspatial linear model; bracket (*), significant difference in paired *t*-test.

Similarly, we interpreted NCEM fits on the deconvoluted Visium lymph node data. We identified a bidirectional dependency of B cells and follicular dendritic cells (FDCs) that is indicative of positive feedback between both cell types in germinal center organization^20^ (Fig. 2a,b and Extended Data Fig. 4c). Similarly, we found evidence for a dependency of mast cells on B cells (Fig. 2b) and a mast cell subcluster associated with niches enriched in B cells (Fig. 2a). The FDC subcluster associated with niches enriched in B cells (cluster 3) showed increased expression of *Cxcl13*, a key chemokine for germinal centers^20^ (Extended Data Fig. 4c), supporting the association of these couplings with germinal centers. We further dissected these couplings based on the gene-wise effects of all senders on one receiver type (‘receiver effect analysis’, Fig. 2c) and of one sender on all receivers (‘sender effect analysis’, Fig. 2d), which contextualizes differential expression results of the FDC–B cell axis (Fig. 2e and Supplementary Data 1). Multiple T cell clusters had a similar effect on B cells in a ‘sender similarity analysis’ (Fig. 2f), in which we correlated the coefficient vectors of sender cell types that correspond to B cell receivers, which demonstrates conservation of cell type identity in the sender profile.

**Extended Data Fig. 8:**
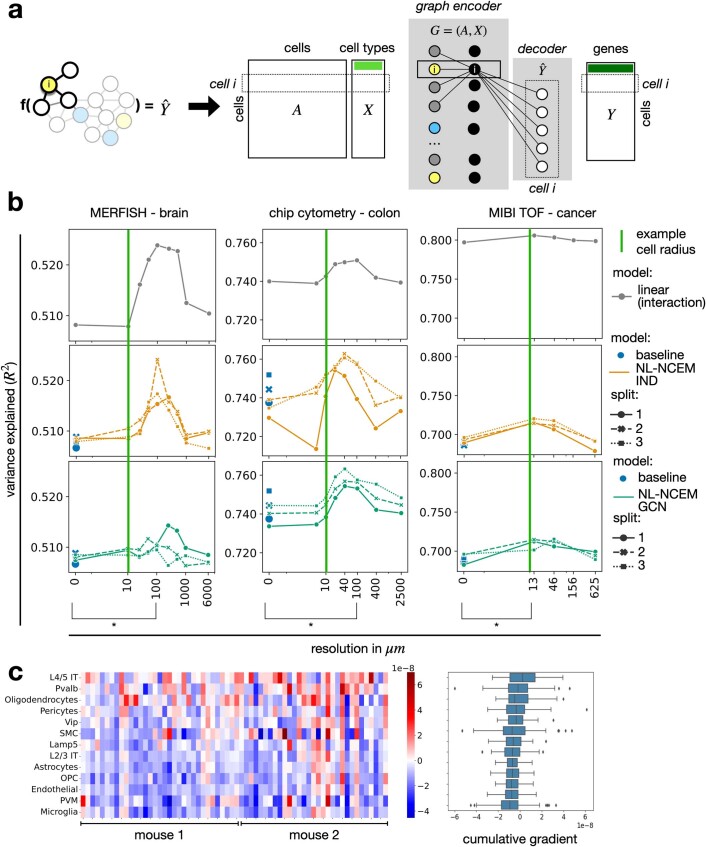
Nonlinear models of spatial dependencies of expression states. (**a**) A node-supervised model in which the label is the expression vector of a cell and the input consists of categorical cell type assignments and a spatial proximity graph. This model can also be viewed as a nonlinear regression model: a local graph embedding of each cell is reconstructed to a cell-wise expression state. The forward pass for a cell *i* is shown. **(b)** Inferred nonlinear spatial dependencies. Shown are the R^2^ values for held-out test data of nonlinear models by resolution in µm with cross validation indicated as point shape and line style and comparatively mean performance of linear model in Fig. 1d. *Linear (interaction) (gray line)*: linear model with interaction effects; *NL*: nonlinear model; *IND*: the graph kernel is an indicator function across cell types in the neighborhood (yellow lines); *GCN*: the graph kernel is a graph convolution, a linear embedding of the cell types in the neighborhood (teal lines); *split (point shapes)*: cross-validation split; bracket (*): significant difference in paired t-test between baseline model and best spatial model with (MERFISH – brain dataset p_IND_ = 0.033, chip cytometry – colon dataset p_GCN_ = 0.026, MIBI-TOF – cancer dataset p_IND_ = 0.005 and p_GCN_ = 0.036). **(c)** Heatmap of cumulative gradients (saliency) of gene expression prediction of L2/3 IT with respect to the input cells, aggregated by the sender cell type clusters, on test data. Shown is a cumulative gradient matrix of L2/3 IT predictions by source cell type and image (n = 64 images). The cumulative absolute gradients are derived from the absolute gradients tensor across each cell’s molecular vector prediction with respect to the cells in the neighborhood (source cells) per image, by taking a sum across the molecular output features and by taking a sum across source cells of the same type. We aggregated these saliency maps per sender-receiver cell type pair as $SALS \in R^{L^\ast L}$, where *L* is the number of distinct cell types in the model. Non-normalized saliencies will show a pattern similar to the contact frequency matrix as cell types with frequent connections will skew the learned importance of cell connections. Therefore, we normalized the saliencies by the absolute number *n*_*ab*_ of occurrences of each cell type pair: $SALS_{ab}^{{{{\mathrm{norm}}}}} = \frac{1}{{n_{ab}}}SALS_{ab}$. For each box in **(c)**, the centerline defines the median, the width of the box is given by the interquartile range (IQR), the whiskers are given by 1.5 * IQR and outliers are given as points beyond the minimum or maximum whisker.

**Extended Data Fig. 9:**
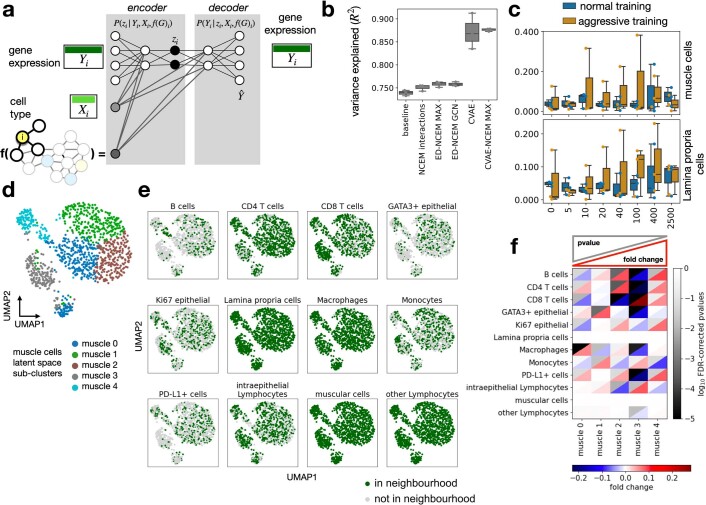
Modeling intrinsic and extrinsic variation in deep latent variable models. **(a)** A node generative network (CVAE–NCEM) is a conditional variational autoencoder in which the condition is not a constant but a graph embedding, which is also learned. The forward pass for a cell *i* through the model is shown. **(b)** Latent variable models improve reconstructive performance. Shown are the R^2^ values of held-out test data based on the forward pass model evaluation from chip cytometry – colon data for linear models, encoder–decoder models, and variational autoencoders for both NCEM and nonspatial models (n=3 cross-validation splits). *baseline*: a nonspatial linear model of gene expression per cell-type; *NCEM interactions*: linear model with interaction effects; *NL*: nonlinear model; *IND*: the graph kernel is an indicator function across cell-types in the neighborhood; *GCN*: the graph kernel is a graph convolution, a linear embedding of the cell-types in the neighborhood. **(c)** Neighborhood transfer performance of NCEM and nonspatial models. Shown is the R^2^ over cells in the test set for models trained on predicting muscular cells and Lamina propria cells for both CVAE and CVAE–NCEMs trained on neighborhoods with different radii with optimization algorithm as color (n=3 cross-validation splits). *Plain*: normal CVAE training; *aggressive*: aggressive encoder training. For each box in **(b, c)**, the centerline defines the median, the height of the box is given by the interquartile range (IQR), the whiskers are given by 1.5 * IQR and outliers are given as points beyond the minimum or maximum whisker. **(d–f)** Latent variables of CVAE–NCEM are confounded with neighborhood conditions. **(d)** UMAP of molecular embedding in the CVAE–NCEM IND latent space of muscular cells in an example image (n = 1,149 cells) with molecular sub-clustering superimposed (muscle 0: n = 315, muscle 1: n = 287, muscle 2: n = 238, muscle 3: n = 183, muscle 4: n = 126)**. (e)** UMAPs of molecular embedding in the CVAE–NCEM IND latent space of all muscle cells in the same image with superimposed binary label of presence of a given cell-type, as defined in the title, in the neighborhood. The underlying neighborhoods were defined at a resolution of 100 µm. **(f)** Heatmap of fold change and false-discovery corrected p-values of cluster enrichment of binary neighborhood labels, where fold changes are the ratio between the relative neighboring source cell-type frequencies per subtype cluster and the overall source cell-type frequency in the image.

In contrast to linear NCEMs, nonlinear NCEMs can account for weighted or higher-order interactions between cell types (Extended Data Fig. 8a). As for linear NCEMs, we found resolution-dependent prediction performance profiles in nonlinear NCEMs (Extended Data Fig. 8b and Methods) and a dependency between L4/5 IT and L2/3 IT cells (Extended Data Fig. 8c and Methods). Notably, the nonlinear models did not outperform linear models in gene expression prediction, which suggests that the spatial dependencies in the given datasets are well described by linear models (Extended Data Fig. 8b). Next, we considered a conditional variational autoencoder (CVAE) version of an NCEM to model cell-intrinsic latent states. We conditioned the distribution over node expression states on a graph embedding of the niche and the cell type (Extended Data Fig. 9a). Even though CVAE–NCEM attained much higher predictive performance in reconstruction tasks (Extended Data Fig. 9b and Supplementary Fig. 7a), these models did not consistently capture spatial dependencies because niche states were represented in latent variables (Extended Data Fig. 9c–f and Supplementary Fig. 7b–e).

**Extended Data Fig. 10:**
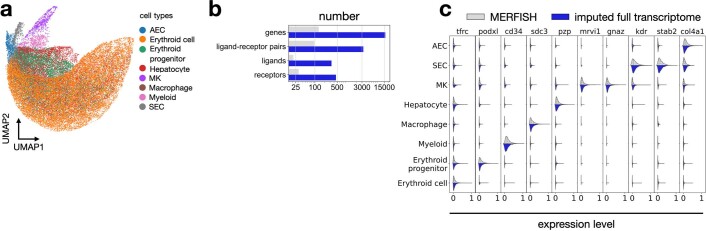
Modeling ligand–receptor signaling with NCEM. **(a)** UMAP of cells in MERFISH – fetal liver data. **(b)** Imputation of MERFISH data with scRNA-seq increases the number of genes that can be modeled with NCEMs, including receptor genes, ligand genes, and ligand–receptor pairs. **(c)** Distribution of selected marker genes that are both observed in scRNA-seq and in MERFISH over cell types.

The interpretation of spatial dependencies inferred on targeted spatial molecular profiling assays is constrained by the limited capture of ligand–receptor pairs. We imputed the cell-wise gene expression in the MERFISH fetal liver data using corresponding scRNA-seq data^21^ (Extended Data Fig. 10). We designed a graph kernel of cell-wise receptor activity to directly model ligand–receptor interactions between neighboring cells (Fig. 2g). The peak predictive performance of the ligand–receptor nonlinear NCEM was much higher compared to the nonlinear NCEM (*R*^2^ of 0.799 and 0.947), demonstrating the increased complexity of the input compared to categorical cell type labels. We observed differential receptor signaling as differential latent neuron activation of *Kit*^11^ in sinusoidal endothelial cells (SECs) depending on their proximity to arterial endothelial cells (AECs) (Supplementary Data 2).

NCEMs are linear and nonlinear graph neural networks and CVAEs that model cell communication events in spatial omics assays (Supplementary Table 2). We identified statistical dependencies between cells on physiologically relevant length scales and interpreted fits based on model parameters. The statistical identifiability of cell type couplings may improve with increased capture of niche heterogeneity, through the inclusion of three-dimensional data, by increasing the number of cells measured and by increasing the variation in the training data through perturbations of niche structure. Uncertainty in segmentation of cells or nuclei can be improved on the level of the spatial measurement^22^ or may be addressed in model extensions. We found that linear NCEMs perform well in the presented tasks and are promising candidates for cell communication inference. The complexity of the graph neural network used in the NCEM defines the complexity of the motifs of cell communication that can be discovered and may be expanded given more complex datasets. CVAE–NCEMs may be used to model cell-intrinsic variation together with niche effects. Similarly, the graph kernels tailored to ligand–receptor signaling presented here provide constrained latent variables that explain extrinsic variation and could be used together with variables for cell-intrinsic variation. Nonlinear NCEMs learn a cellular representation within the spatial graph^23^, and we demonstrated that these representations can model niches and may be exploited for unsupervised analysis of tissue structures.

## Methods

### Data

#### Fetal liver (MERFISH)

Lu et al.^11^ measured wild type (WT) and Tet^2−/−^ fetal livers with MERFISH in 140 (WT) and 195 (Tet^2−/−^) images across four WT fetal livers at E14.5 and two Tet^2−/−^ knockout fetal livers at E14.5, with 132 genes observed in 40,864 (WT) and 54,970 (Tet^2−/−^) cells. We used cell types as originally annotated by Lu et al.: AEC, erythroid cell, erythroid progenitor, hepatocyte, megakaryocyte, macrophage, myeloid and SEC. We removed cells with an unknown label from the dataset. We scaled model outputs by the node size in the respective output layer of each model class to mitigate count noise (Supplementary Fig. 8f).

#### Brain (MERFISH)

Zhang et al.^12^ measured mouse primary motor cortex with MERFISH in 64 images across two mice, with 254 genes observed in 284,098 cells. We used the cell types originally annotated by Zhang et al.: astrocytes, endothelial, L2/3 IT neurons, L4/5 IT, L5/6 near-projecting neurons, L5 IT, L5 pyramidal tract neurons, L6 cortico-thalamic projection neurons, L6 IT, L6 IT Car3, L6b, Lamp5, microglia, oligodendrocyte precursor cells, oligodendrocytes, perivascular macrophages, pericytes, parvalbumin, smooth muscle cells, Sncg, somatostatin (Sst), Sst Chodl, vascular leptomeningeal cells, vasoactive intestinal polypeptide, and other cells, where L identifies the layer (L1–L6) of the distinctive laminar structure based on cytoarchitectural features (Extended Data Fig. 2a). Parvalbumin, Sst, vasoactive intestinal polypeptide, Sncg and Lamp5 define five subclasses of GABAergic cells. We removed cells labeled as ‘other’ from the dataset. We used an identifier for the respective mouse as domain information.

#### Colon (chip cytometry)

Jarosch et al.^16^ measured an inflamed colon with chip cytometry in two images from one patient, with 19 genes observed in 11,321 cells. We used the cell types originally annotated by Jarosch et al.: B cells, CD4 T cells, CD8 T cells, GATA3^+^ epithelial, Ki67 high epithelial, Ki67 low epithelial, lamina propria cells, macrophages, monocytes, PD-L1^+^ cells, intraepithelial lymphocytes, muscular cells and other lymphocytes (Extended Data Fig. 2a). We coarsened the cell type annotation by combining Ki67 high epithelial and Ki67 low epithelial to a joined annotation of Ki67 epithelial. We log-transformed the gene expression values for use in the analyses presented here to mitigate count noise (Supplementary Fig. 8b).

#### Cancer (MIBI-TOF)

Hartmann et al.^14^ measured colorectal carcinoma and healthy adjacent tissue with MIBI-TOF in 58 images across four individuals, with 36 genes observed in 63,747 cells. We used the cell types originally annotated by Hartmann et al.: endothelial, epithelial, fibroblast, CD11c myeloid, CD68 myeloid, CD4 T cells, CD8 T cells and other immune cells (Extended Data Fig. 2a). The cohort in this dataset includes two patients with colorectal carcinoma and two healthy controls. We scaled the model outputs by cell-wise size factors.

#### Tonsils (MELC)

Pascual-Reguant et al.^15^ measured tonsils from patients undergoing tonsillectomy with multiepitope ligand cartography (MELC), an immunohistochemistry approach, in one image across one patient, with 51 genes observed in 9,512 cells. We used the cell types originally annotated by Pascual-Reguant et al.: B cells, endothelial cells, innate lymphoid cell (ILC), monocytes/macrophages/dendritic cells (DC), natural killer (NK) cells, plasma cells, T cytotoxic cells, T helper cells (Extended Data Fig. 2a). We removed cells labeled as ‘other*’* from the dataset.

#### Cancer (CODEX)

Schürch et al.^13^ measured advanced-stage colorectal cancer with CODEX in 140 images across 35 patients, with 57 genes observed in 272,266 cells. We used the cell types originally annotated by Schürch et al.: B cells, CD11b^+^ monocytes, CD11c^+^ dendritic cells, CD11b^+^ CD68^+^ macrophages, CD163^+^ macrophages, CD68^+^ macrophages, CD68^+^ macrophages GzmB^+^, CD68^+^ CD163^+^ macrophages, CD3^+^ T cells, CD4^+^ T cells, CD4^+^ T cells CD45RO^+^, CD4^+^ T cells GATA3^+^, CD8^+^ T cells, NK cells, T regs, adipocytes, dirt, granulocytes, immune cells, immune cells/vasculature, lymphatics, nerves, plasma cells, smooth muscle, stroma, tumor cells, tumor cells/immune cells and vasculature (Extended Data Fig. 2a). We removed cells with an annotation of dirt or an undefined label from the dataset. We merged the macrophage subclusters (CD11b^+^ CD68^+^ macrophages, CD163^+^ macrophages, CD68^+^ macrophages, CD68^+^ macrophages GzmB^+^ and CD68^+^ CD163^+^ macrophages) and the CD4^+^ T cell subclusters (CD4^+^ T cells, CD4^+^ T cells CD45RO^+^ and CD4^+^ T cells GATA3^+^). We scaled model outputs by the node size in the respective output layer of each model class to mitigate count noise (Supplementary Fig. 8e).

#### Lymph node (Visium)

We performed deconvolution with cell2location^19^ on a 10x Visium lymph node dataset based on a scRNA-seq dataset from the same tissue as previously described^19^. The cell type labels used for deconvolution were B cells, DC, endothelial, follicular dendritic cells, ILC, macrophage, mast, monocytes, NK, natural killer T cells (NKT), CD4 T cells, CD8 T cells, T cells (TIM3), T follicular regulatory cells _fr_, T regulatory cells _reg_) and vascular smooth muscle cells.

#### Dataset partitions

We randomly selected 10% of all nodes across all images and patients as the test set. From the remaining nodes, 10% of all nodes are selected as the validation set.

### MERFISH-scRNA-seq integration

We integrated scRNA-seq with MERFISH data to impute the full gene expression in the spatial graph of cells measured in MERFISH. We performed this integration between the MERFISH fetal liver (WT) data and scRNA-seq of E14.5 whole fetal liver cells sequenced by 10x Genomics platform^11^, which is available as GSE172127 on GEO. The scRNA-seq dataset contains 9,448 cells across 28,692 features. We performed quality control and removed cells with fewer than 500 detected genes, genes expressed in less than three cells and cells with more than 10% of the transcripts coming from mitochondrial genes from the dataset. We applied Tangram^21^ to generate a spatially resolved representation of the scRNA-seq fetal liver dataset^21^. We used 131 out of 132 genes from the MERFISH fetal liver (WT) data, which were both present in the MERFISH and the scRNA-seq dataset, to perform the mapping.

### Variance decomposition into inter- and intra-cell-type variance

The variance of a single-cell resolved dataset can be decomposed into intercell-type variance, intracell-type variance and gene variance. The gene variance is independent of cell type definitions and can therefore be considered separately from intra- and inter-cell-type variance:

1 $$\begin{array}{l}\mathop {\sum}\limits_i^N {\mathop {\sum}\limits_j^J {\left( {y_{i,j} - {\bar {\text {y}}}} \right)^2} }\\ = \underbrace {\mathop {\sum}\nolimits_i^N {\mathop {\sum}\nolimits_j^J {\left( {y_{i,j} - \bar y_{k(i),j}} \right)^2} } }_{{\mathrm{intracell-}}\,{\mathrm{type}}\,{\mathrm{variance}}} + \underbrace {\mathop {\sum}\nolimits_i^N {\mathop {\sum}\nolimits_j^J {\left( {\bar y_{k(i),j} - \bar y_j} \right)^2} } }_{{\mathrm{intercell-}}\,{\mathrm{type}}\,{\mathrm{variance}}} + \underbrace {\mathop {\sum}\nolimits_i^N {\mathop {\sum}\nolimits_j^J {\left( {\bar y_j - {\bar {\text{y}}}} \right)^2} } }_{{\mathrm{gene}}\,{\mathrm{variance}}}\end{array}$$

where *y*_*i*,*j*_ is the gene expression of cell *i* out of *N* and gene *j* out of *J*, $\bar x_{k,j}$ is the mean expression of each gene *j* in each cell type *k*, *k*(*i*) is the cell type of cell *i*, ${\bar {\text{y}}}_j$ is the mean expression of each gene *j* and $\bar y$ is the mean of the dataset.

### Simulations

#### Segmentation errors

We simulated segmentation errors in which the segment boundary between two neighboring cells is misplaced (Extended Data Fig. 3f,g). We selected a fraction of cells (10% or 50%) in the MERFISH fetal liver data at random, selecting one neighbor at random for each selected cell, and transferred a fraction of the total molecular abundance vector of the selected cell to its neighbor.

#### Spatial dependencies

We simulated single-cell resolved spatial data from scratch by using the cell graph from the chip cytometry colon data (Extended Data Fig. 2) and the simulated node-wise gene expression vectors. We modeled cell types using the number of genes originally defined in the dataset and drew a mean expression value for each gene from a uniform distribution between 0 and 10. We considered two scenarios of dependencies between cells: (1) a dataset without spatial dependencies in which all cells are drawn from one cell type and are independent and identically distributed and (2) a dataset with spatial dependencies in which cells belong to either one of two cell types, where we introduced dependencies on the presence of the respective other cell type in the neighborhood in 50% of the genes, with strong effect sizes drawn from a uniform distribution between 4 and 6. We fitted NCEMs, Misty^9^ and SVCA^4^ on both simulated datasets (Supplementary Fig. 2). The simulated images contained over 4,500 cells per image. To reduce the runtime for SVCA for these samples we cropped both images in the lower-right corner to create images with approximately 850 cells each.

### Models

The inputs to NCEMs are (1) a gene expression matrix $Y \in R^{N \times J}$ where *N* is the number of cells and *J* is the number of genes, (2) a matrix of observed cell types $X^{\tt{l}} \in R^{N \times L}$ where *L* is the number of unique cell type labels and (3) a matrix specifying the batch assignments $X^{\tt{c}} \in R^{N \times C}$ of *C* distinct batches or domains, such as images or patients. We denote the adjacency matrix of connected cells as $A \in R^{N \times N}$, which is calculated based on the spatial proximity of cells per image. For linear models and models with an indicator aggregator, we used a binary adjacency matrix *A*_*ij*_ = 1 if $d(x_a,x_b) \le \delta _{{\mathrm{max}}}$ where $d( \cdot , \cdot )$ describes the euclidean distance between nodes *a*, *b* ∈ *N* in space and *δ*_max_ is the neighborhood size (resolution), and *A*_*ij*_ = 0 otherwise. For models using graph convolutions, we normalized *A* such that all rows sum to one: *D*^−1^_*A*_ where *D* is the diagonal node degree matrix. The output of NCEMs is $\hat{Y} \in R^{N \times J}$, a reconstruction of the gene expression matrix. In selected datasets, we applied size factor scaling to the network output *Y* using the size factors ${{{\mathrm{sf}}}}_i = \frac{{\mathop {\sum}\nolimits_j^J {y_{ij}} }}{{\frac{1}{N}\mathop {\sum}\nolimits_{i^\prime }^N {\mathop {\sum}\nolimits_{j^\prime }^J {y_{i^\prime j^\prime }} } }}$. The global data handling per dataset is reported in Supplementary Table 3, model hyperparameters for linear models are reported in Supplementary Table 4, and the parameters for nonlinear and CVAE models in Supplementary Table 5.

#### Loss functions

We use a Gaussian log-likelihood, ll, loss as an optimization objective for linear and nonlinear models with ${\mathrm{ll}}(y) = \frac{1}{{N * J}}\mathop {\sum}\limits_i^N {\mathop {\sum}\limits_j^J {( { - {\mathrm{log}}\left( {\sqrt {2\pi } \sigma _j} \right) - 0.5\frac{{\left( {y_{ij} - \hat{y}_{ij}} \right)^2}}{{\sigma _j^2}}} )} }$ over cells *i* and genes *j*, where *σ*_*j*_ is the predicted standard deviation of a gene (Supplementary Fig. 8). The loss function of CVAE model is the negative data log-likelihood in addition to the Kullback–Leibler divergence between the variation posterior *q*_*ϕ*_(*z*) and the prior *p*(*z*) on the latent variables: ${\mathrm{ll}}_{{{{\mathrm{CVAE}}}}} = - {\mathrm{ll}}(y) + D_{{{{\mathrm{KL}}}}}\left( {q_\phi (z)||p(z)} \right)$.

#### Optimization

We ran grid searches to find the optimal set of hyperparameters for each dataset where the batch size is the number of images per dataset. We selected the number of nodes evaluated per image per batch to improve convergence. We trained all models with the Adam optimizer: linear models with 0.05, the remaining models with multiple learning rates of {0.5, 0.05, 0.005}. Additionally, we used a learning scheduler on the validation loss with a patience of 20 epochs, which reduces the learning rate by a factor of 0.5, so ${\mathrm{lr}}_{\mathrm{new}} = {\mathrm{lr}} \ast 0.5$ and early stopping with a patience of 100 epochs. The exact description of all grid searches in code are supplied in the benchmarking repository (Code Availability). We trained linear models for hypothesis testing using ordinary least squares estimators on the full dataset.

#### Linear NCEM

The linear nonspatial baseline model infers a reconstruction *Ŷ* from a node’s cell type and respective domain information via  ﻿Ŷ= *X*^*D*^*β*, where *X*^*D*^ is the design matrix and $\beta \in R^{(L + C) \times J}$ are the parameters learned by the model. The design matrix of nonspatial baseline models is given by $X^{\tt{D}} = (X^{\tt{l}},X^{\tt{c}}) \in R^{N \times (L + C)}$. The spatial counterpart model, the linear NCEM, has access to an additional spatial sender–receiver interaction matrix. First, we computed the binary sender cell presence in the neighborhood of each cell $X^{\tt{S}} = 1_{(AX^l > 0)} \in \{ 0,1\} ^{N \times L}$, where 1_(·)_ represents an indicator function. To generate a matrix representation of sender–receiver cell interactions, we compute the interaction terms between the cell type of the index cell and the presence of each cell type in its neighborhood as the outer product between *X*^*l*^ and *X*^*S*^. The resulting interaction matrix is $X^{\tt{TS}} \in \{ 0,1\} ^{N \times L^2}$, and the design matrix for the linear model with interaction terms is given by $X^{\tt{D}} = (X^{\tt{l}},X^{\tt{TS}},X^{\tt{c}}) \in R^{N \times (L + L^2 + C)}$. This design matrix can be related to a graph neural network: *X*^*l*^ and *X*^*c*^ are node-wise condition vectors that can be appended to a local graph embedding centered on an index cell, and *X*^*TS*^ is equivalent to an outer product of the one-hot-encoded representation of an index cell with the projection obtained from a single-layer graph neural network that embeds one-encoded cell type feature vectors with a feature-wise max pooling operator across the neighborhood without the index cell. This projection is a cell-type-dimensional indicator for the presence of each cell type in the neighborhood. Linear NCEMs perform parameter inference on Ŷ = *X*^*D*^*β* where $\beta \in R^{(L + L^2 + C) \times J}$. We also considered an NCEM without interaction terms which does not have receiver-specific sender effects but only global sender effects, which account for the presence of senders in the niche via $X^{\tt{D}} = (X^{\tt{l}},X^{\tt{S}},X^{\tt{c}}) \in R^{N \times (L + L + C)}$. We evaluated significance of coefficients corresponding to the interaction matrix *X*^*TS*^ with a Wald test.

#### Linear NCEM for deconvoluted spot transcriptomics

The baseline model is the same as for the standard linear NCEM. The corresponding NCEM treats the spot as a neighborhood and uses the deconvoluted cell type abundances per spot $X^{\tt{F}} \in R^{(N*L)xL}$ as a vector-shaped neighborhood summary, replacing a kernel on a graph. Note that (N*L) is the number of spots times the number of cell types: this model treats every type- and spot-wise gene expression vector, a result of the deconvolution, as an observation. The overall design matrix of the linear model includes the interaction between the target cell type and the spot composition, and spot-wise covariates: $X^{\tt{D}} \in R^{(N*L) \times (L + L^2 + C)}$. Note that here, the spot composition is the same for all L gene expression prediction problems per spot. As for the linear NCEM, we again fit a linear model to this design matrix to predict deconvoluted gene expression. One can define a corresponding nonlinear model that uses the deconvoluted cell type abundances per spot $X^{\tt{F}} \in R^{N \times L}$ as a vector-shaped node feature space. Note that *N* is the number of spots in this nonlinear model. These feature vectors can be connected based on spot proximity in a graph embedding of spots $f_{{{{\mathrm{enc}}}}}:q_\phi (z_s|g(A,X^{\tt{l}})_s)$. The cell-type-wise gene expression decoder for spot *s* and cell type *k* is then $f_{{{{\mathrm{dec}}}}}:p_\theta (Y_{sk}|z_s,X^{\tt{k}},X_s^{\tt{c}})$*,* where *X*^*k*^ is a one-hot embedding of the cell type *k*.

#### Nonlinear NCEM

NCEMs include nonlinear models that encode the neighborhood through a graph neural network (NL-NCEM) and decode expression vectors. The corresponding nonspatial baseline model is a nonlinear model (NL) that predicts expression from cell type and graph-level predictors. A local graph embedding is given by $f_{{{{\mathrm{enc}}}}}:q_\phi (z_i|X_i^l,g(A,X^{\tt{l}})_i,X_i^{\tt{c}})$, which encodes the cell type labels *X*^*l*^, some graph-level predictors *X*^*c*^ and the local graph embedding *g*(*A*, *X*^*l*^), based on the adjacency matrix *A*, into a latent state *z*. The latent state of cell *i* is input to a fully connected layer stack given by $f_{{{{\mathrm{dec}}}}}:p_\theta (Y_i|z_i,X_i^{\tt{l}},X_i^{\tt{c}})$. If one uses an indicator embedding function as described in the section Linear NCEM and all hidden layers are removed from the NL-NCEM, a single linear transformation of the input remains, which is equivalent to the linear NCEM. Alternatively, *g*(*A*, *X*^*l*^) can be a graph embedding learned by a graph-convolutional network (GCN)^5^. A one-layer GCN is given by $g(A,X^l) = {\it{{\mathrm{softmax}}}}( {ReLU(\bar AX^lW)} )$, where $W \in R^{L \times H}$ is a weight matrix, *H* is the dimension of the learned node representation and $\bar A$ is the normalized adjacency matrix.

#### Ligand–receptor NCEM (NL-NCEM-LR)

Here we consider a specific NL-NCEM with a tailored graph kernel. This graph kernel embeds each cell into a receptor dimensional latent space *z* based on the receptor gene expression on the index cell, ligand gene expression on neighboring cells, and the adjacency matrix *A*, which encodes the set of neighbors *MN* of cell *i*: $f_{{{{\mathrm{enc}}}}}:z_{ik} = g(A_i,Y_{i,r(k)},Y_{:,l(k)}) = \mathop {\sum}\nolimits_m^M {f_R(Y_{i,r(k)}) * f_L\left( {Y_{m,r(k)}} \right)}$. Here, *r*(*k*) and *l*(*k*) encode the gene index of receptor and ligand that correspond to ligand*–*receptor pair *k*. The latent state and graph-level predictors *X*^*c*^ are input to a fully connected layer stack that decodes gene expression $f_{{{{\mathrm{dec}}}}}:p_\theta (Y_i|z_i,X^{\tt{c}})$. The corresponding nonspatial baseline is a nonlinear model (NL) that receives receptor expression of the index cell as bottleneck activation and has the same decoder. This baseline model is not nested in the nonlinear ligand–receptor NCEM (NL-NCEM-LR) but models a baseline which imputes all genes’ expression based on ligand gene expression within the cell.

#### Conditional variational autoencoder NCEM

A conditional variational autoencoder NCEM (CVAE–NCEM) learns a distribution over node states *Y* based on a node-wise latent space *z*. The nonspatial CVAE null model contains the cell type and graph-level predictors as a condition in the variational posterior and the likelihood model. In CVAE–NCEM, the conditions are the cell type labels *X*^*l*^, some graph-level predictors *X*^*c*^ and the local graph embedding *g*(*A*, *X*^*l*^). The encoder is given by $f_{{\it{{\mathrm{enc}}}}}:q_\phi ( {z_i|Y_i,X_i^{\tt{l}},g(A,X^{\tt{l}})_i,X_i^{\tt{c}}} )$ and the decoder is defined by $f_{{{{\mathrm{dec}}}}}:p_\theta ( {Y_i|z_i,X_i^{\tt{l}},g(A,X^{\tt{l}})_i,X_i^{\tt{c}}} )$. A CVAE–NCEM for a full dataset depends on both the niche and the type of the cell itself. This setting presents the challenge of encountering a nonidentifiability between variance attributed to latent variables, cell type conditions and neighborhood context. In this study, we consider the CVAE–NCEM trained on the molecular vectors of a single target cell type as a function of the full neighborhood context to remove the nonidentifiability with respect to cell type variation and focus on the nonidentifiability between latent variables and neighborhoods.

### Model evaluation

We evaluated model performance using the coefficient of determination: $R_i^2 = 1 - \frac{{\mathop {\sum}\nolimits_j^J {\left( {y_{ij} - \hat{y}_{ij}} \right)^2} }}{{\mathop {\sum}\nolimits_j^J {\left( {y_{ij} - \bar y_{ij}} \right)^2} }}$ for cells *i* and over genes *j*. We selected the best performing models based on *R*^2^ on a validation dataset and showed this metric evaluated on test data in the manuscript. The performance of CVAEs is additionally assessed in style transfer tasks. In style transfer, the gene expression state and neighborhood of a reference node *a* from the source domain is encoded to estimate the latent states of this node. This latent representation is then decoded to the target domain of cell *b*, which implies conditioning the decoding on the target neighborhood:

2 $$z_a \sim q_\phi \left( {\bf{z}|Y_a,X_a^{\tt{l}},g(A,X^{\tt{l}})_a,X_a^{\tt{c}}} \right)$$

3 $$\hat{Y}_b = p_{\theta} \left( {\mathbf{{z}_a},{\mathbf{X}}_b^{\tt{l}},g({\mathbf{A}},{\mathbf{X}}^{\tt{l}})_b,{\mathbf{X}}_b^{\tt{c}}} \right)$$

where *a*, *b* are cell indices, *q*_*ϕ*_ is the amortized variational posterior and *p*_*θ*_ is the decoder network. See also Conditional variational autoencoder NCEM for details on the notation.

### Unsupervised analysis

We used uniform manifold approximation and projection (UMAP) to embed the cells in two dimensions for visualization of high-dimensional data.

We computed the UMAP of B cell, FDC and mast cell substates (Fig. 2a) based on 50 principal components (PCs) and *k* = 500. We computed the UMAP of the scRNA-seq reference dataset of lymph nodes (Extended Data Fig. 4b) based on 50 PCs with *k* = 100. We computed the UMAP of the MERFISH brain data^12^ matrix (Extended Data Fig. 5a) based on the first 35 PCs and the *k*-nearest neighbor graph with *k* = 10. We computed the UMAP of L2/3 IT neurons in slice 153 (Extended Data Fig. 5a) and slice 162 (Supplementary Fig. 5a) of the MERFISH brain dataset based on the first 40 PCs with *k* = 40 and performed Louvain community detection using Scanpy^17^ to define stable L2/3 IT substates. We computed UMAPs of CD8 T cells in area 1 in the chip cytometry dataset (Extended Data Fig. 6) based on the gene expression matrix directly and *k* = 22, and UMAPs of CD8 T cells in image 1, 5, 8 and 16 of the MIBI-TOF cancer dataset (Extended Data Fig. 7) based on the gene expression directly and *k* = 60. We performed Louvain community detection of the latent space in CVAE and CVAE–NCEM IND models (Extended Data Fig. 9d,e and Supplementary Fig. 7c,d) based on the latent space using *k* = 80 for the MERFISH brain dataset and *k* = 250 for the chip cytometry colon dataset.

We performed cluster enrichment with Fisher’s exact test. Each contingency table is composed of two categorical variables. The first variable describes the binary assignment of cells to one L2/3 IT subcluster. The second variable describes the presence of a source cell type in their neighborhood. We performed Benjamini and Hochberg false discovery rate correction (FDR) of cluster enrichment *P* values. A similar approach was used for the cluster enrichment analysis of CD8 T cells in the chip cytometry colon and the MIBI-TOF cancer datasets.

### Type coupling analysis

We performed type coupling analysis, sender effect and receiver effect analysis based on a Wald test on the parameters estimates of linear NCEM obtained on the full dataset as ordinary least squares estimates. We performed FDR-correction of the resulting *P* values using the Benjamini-Hochberg correction method. The coupling measure between sender and receiver cells is the L1-norm of coefficients of significant coefficients that correspond to the specific receiver–sender interaction term in the linear model, or the number of differentially expressed genes. The sender effect and receiver effect analysis consists of the set of coefficients and their significance for a particular sender and receiver, respectively. The sender similarity analysis is a hierarchical clustering of the Pearson product-moment correlation coefficients of coefficient vectors of sender cell types for one defined receiver cell type.

### Differential receptor activity in NL-NCEM-LR

We used a *t*-test to obtain a ranking for highly differential receptor signaling in SEC depending on the presence of neighboring AEC. We used the neighborhood size that corresponded to the best performing resolution of the NL-NCEM-LR model.

### Subsampling robustness analysis

We randomly subsampled the spatial transcriptomics spots from the Visium lymph node data to 5%, 25%, 50% and 75% of all spots across three cross validations. We deconvoluted the resulting subsampled slide with cell2location and used this inferred spot composition as input to the NCEM type coupling analysis for spot-transcriptomic data. In order to assess the robustness with respect to identified putative dependencies, we computed the *R*^2^ between the inferred coefficient vectors over genes for each cell type pair between the fit to the complete data and the fit to the subsampled slide.

### CellPhoneDB and NicheNet

We inferred putatively communicating ligand–receptor pairs in lymph nodes using CellPhoneDB as implemented in squidpy^7^ on scRNA-seq data^24^ with *n* = 53,275 cells on the 10,000 most variable genes. We quantified sender–receiver interactions as the number of significant ligand–receptor pairs at an FDR-corrected *P* value of 0.05. Additionally, we considered the presence of nonzero expression of cognate ligand–receptor pairs (Extended Data Fig. 4e). We performed the CellPhoneDB analysis shown in Supplementary Fig. 6 based on *n* = 1,000 permutations. Additionally, we used randomly subsampled data for the analysis of MERFISH brain^12^ 10% with *n* = 27,655, MIBI TOF cancer^14^ 40% with *n* = 25,498 and CODEX cancer^13^ 10% with *n* = 25,186.

We defined the 5,000 most variable genes per receiver cell type as target genes in a NicheNet analysis. For the following cell types, we limited the number of highly variable genes to the number given in brackets depending on the respective intracell-type heterogeneity: DC (500), endothelial (1,500), erythrocyte (250), HSC (1,000), macrophages (4,000), mast (1,000), monoctyes (2,000), myeloid (2,000), neutrophil (400), stromal cells (1,500) and T T_reg_ (3,000). We defined all remaining genes as background genes for NicheNet. We selected the top-100-ranked ligands from NicheNet and thresholded the putative ligands to be expressed in at least 5% of all sender cells.

### Reporting summary

Further information on research design is available in the Nature Research Reporting Summary linked to this article.

## Online content

Any methods, additional references, Nature Research reporting summaries, source data, extended data, supplementary information, acknowledgements, peer review information; details of author contributions and competing interests; and statements of data and code availability are available at 10.1038/s41587-022-01467-z.

## Supplementary information

Supplementary Information Supplementary Figs. 1–8 and Tables 1–5.

Reporting Summary

Supplementary Data 1 Effect of FDC on B cells in the Visium lymph node dataset presented in the sender effect and receiver effect analysis.

Supplementary Data 2 Differential latent unit activity on MERFISH fetal liver (wild type, imputed) dataset of ligand–receptor nonlinear NCEM, between SECs with and without AECs in the neighborhood. Shown is a *t*-test between the two sets of cells for each latent unit which each correspond to a ligand–receptor pair.

## Data Availability

The MERFISH fetal liver^11^, MERFISH brain^12^, MIBI TOF cancer^14^, MELC tonsils^15^, CODEX cancer^13^, chip cytometry colon^16^ and Visium lymph node^19^ datasets are publicly available (Methods).

## Extended data

are available for this paper at 10.1038/s41587-022-01467-z.

## Code availability

All models described here are implemented in a Python package available at https://github.com/theislab/ncem. All benchmarking and analysis codes are provided at https://github.com/theislab/ncem_benchmarks. Tutorials for model usage are available from https://github.com/theislab/ncem_tutorials.
