# Supplementary material for: Modeling intercellular communication in tissues using spatial graphs of cells
Source: Nat Biotechnol. 2022 Oct 27;41(3):332–6. doi: 10.1038/s41587-022-01467-z (PMC10017508; doi:10.1038/s41587-022-01467-z)
Supplement: Supplementary file 1 — Supplementary Figs. 1–8 and Tables 1–5. [file 41587_2022_1467_MOESM1_ESM.pdf]

nature portfolio

https://doi.org/10.1038/s41587-022-01467-z

Supplementary information

Modeling intercellular communication in tissues using spatial graphs of cells

In the format provided by the
authors and unedited

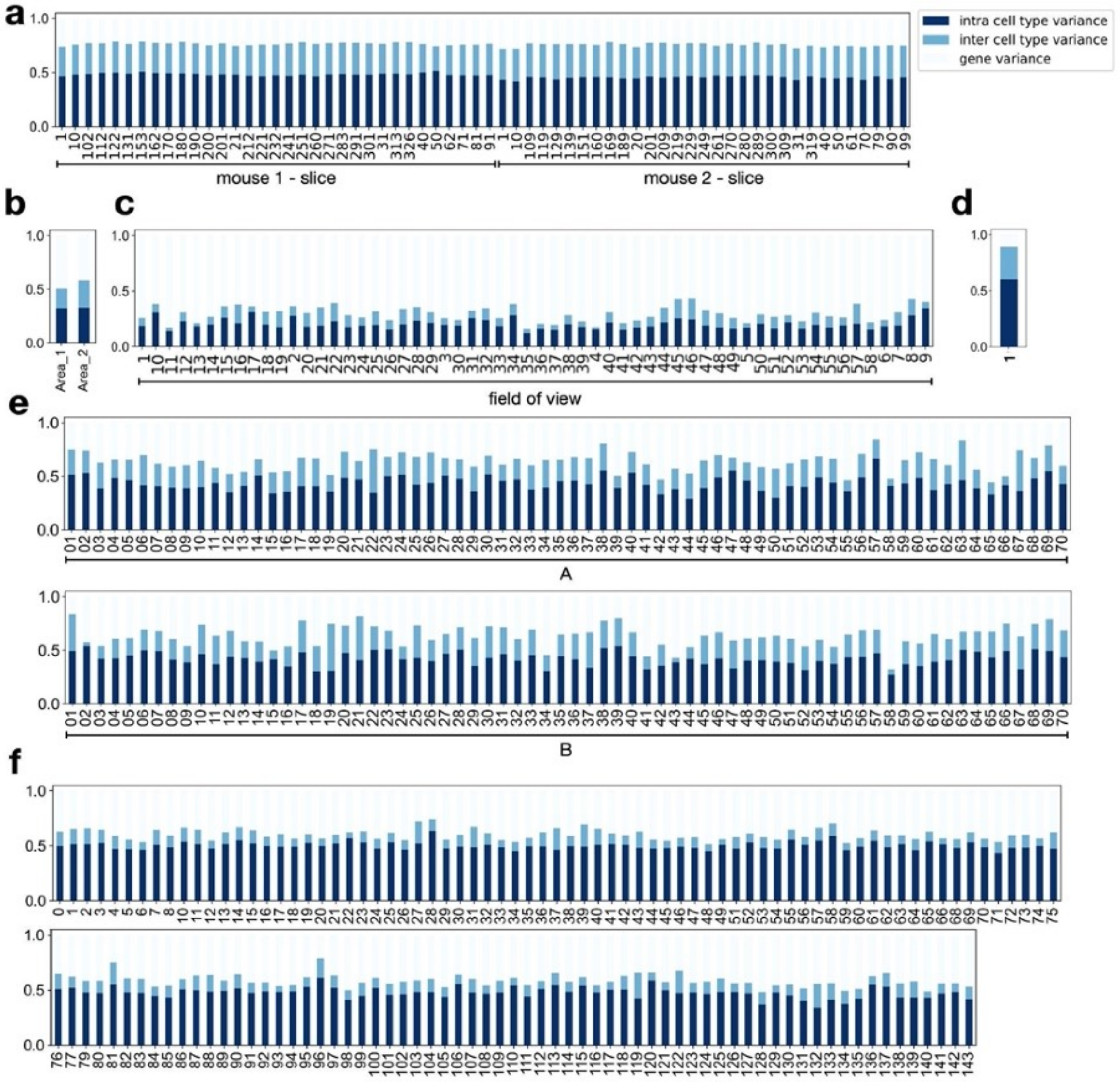

Supp. Fig. 1: Variance decomposition in spatial omics datasets. MERFISH – brain (mean intra cell type variance: 47%, mean inter cell type variance: 29%, mean gene variance: 24%) **(a)**, chip cytometry – colon (mean intra cell type variance: 33%, mean inter cell type variance: 22%, mean gene variance: 45%) **(b)**, MIBI TOF – cancer (mean intra cell type variance: 20%, mean inter cell type variance: 10%, mean gene variance: 70%) **(c)**, MELC – tonsils (mean intra cell type variance: 60%, mean inter cell type variance: 29%, mean gene variance: 11%) **(d)** and CODEX – cancer dataset (mean intra cell type variance: 43%, mean inter cell type variance: 21%, mean gene variance: 36%) with images ordered by tissue microarrays (*A, B*) **(e)**, MERFISH - wild type fetal liver (mean intra cell type variance: 49%, mean inter cell type variance: 11%, mean gene variance: 40%) **(f)**.

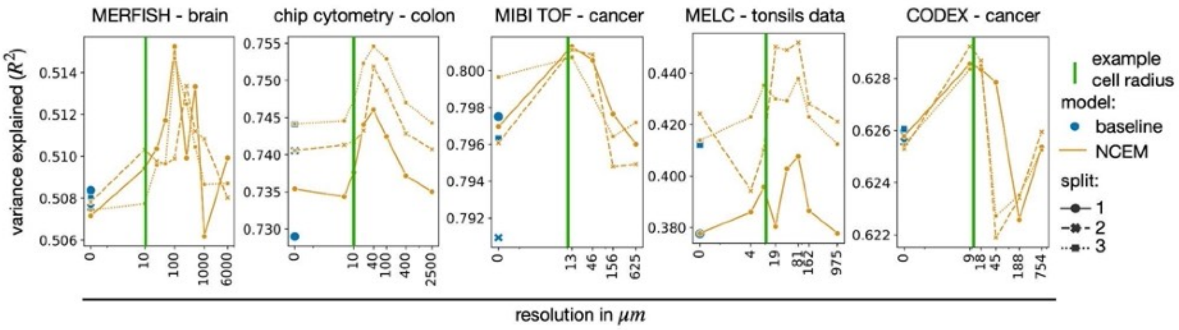

Supp. Fig. 2: Linear models for spatial cell state dependencies. Linear models without receiver-sender interaction terms (Online Methods) capture neighborhood dependencies in spatially resolved single-cell data. Shown are R^2 for held-out test data of linear models by resolution in µm with cross validation indicated as point shape and line style. The underlying linear models are parameterised with sender cell-type-specific parameters. *example cell radius (green line)*: Example length scale of a cell, here chosen as 10 µm; *baseline (blue dot)*: a nonspatial linear model of gene expression per cell-type; *NCEM*: linear NCEM.

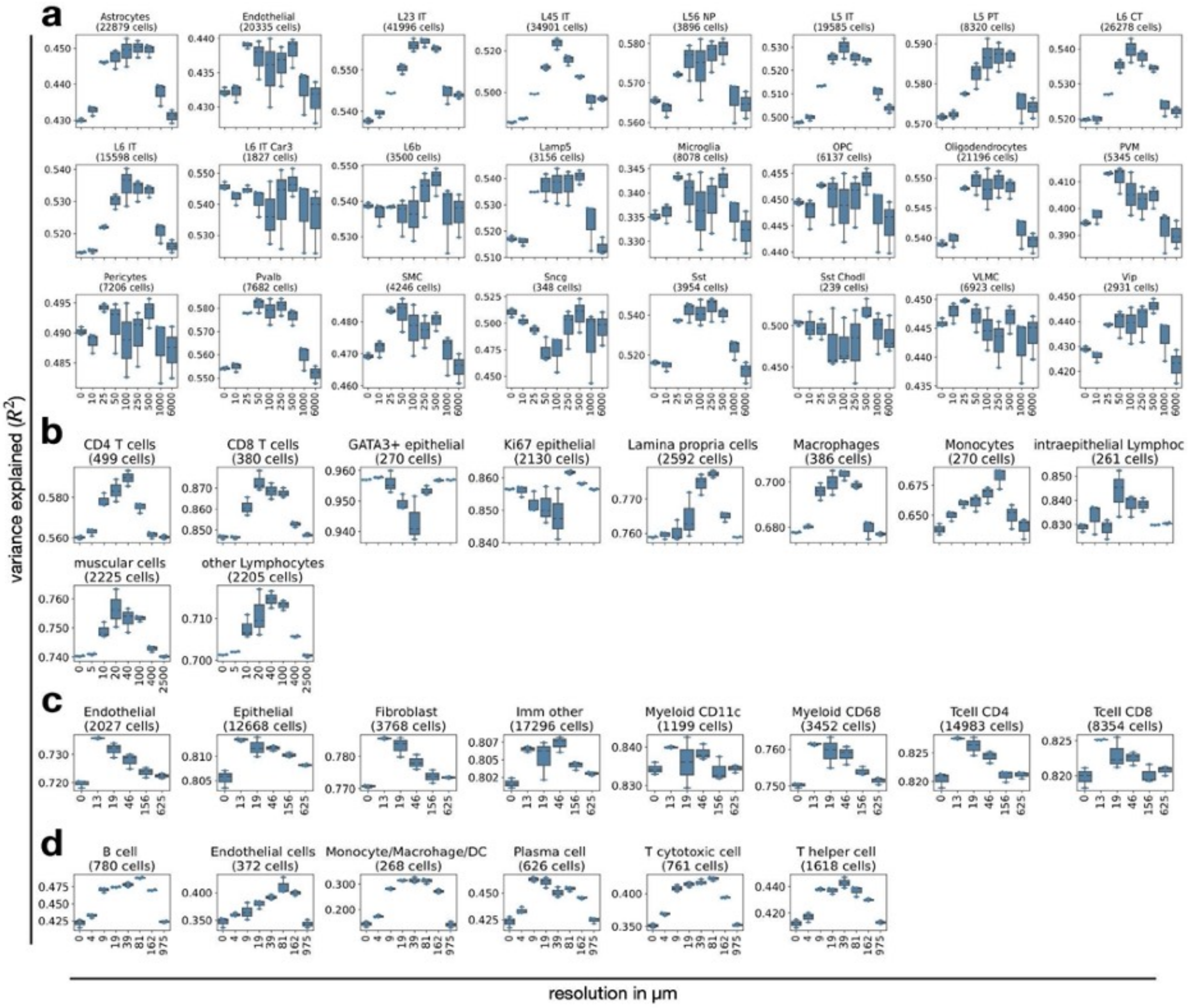

Supp. Fig. 3: Length scales of dependencies for different target cell-types. Shown are R^2 for held-out test data of linear models by resolution in µm for different predicted cell-types for MERFISH – brain data **(a)**, chip cytometry – colon data **(b)**, MIBI TOF – cancer **(c),** MELC – tonsils **(d)** where each boxplot corresponds to a three-fold cross validation (n is the number of cells per data set partition as indicated in the each plot title). For each box in **(b-d)**, the centerline defines the median, the height of the box is given by the interquartile range (IQR), the whiskers are given by 1.5 * IQR and outliers are given as points beyond the minimum or maximum whisker.

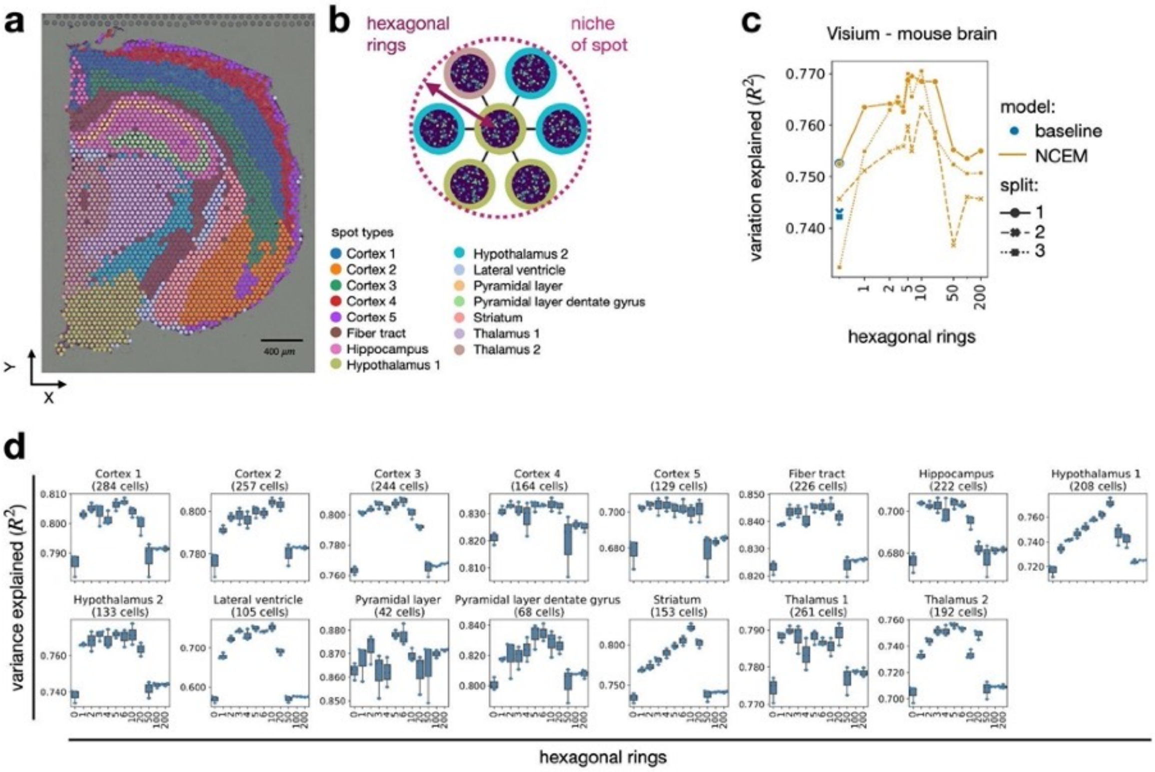

Supp. Fig. 4: Modeling cross-niche dependencies in spot transcriptomics data. (a) Cluster labels of a clustering of spots measured with spot transcriptomics in mouse brain. **(b)** Structure of a neighborhood of niches (spots) to model with NCEM in spot transcriptomics. **(c)** Linear models capture spot-to-spot dependencies in spot-transcriptomics data. Shown are the R^2 values between predicted expression vectors and observed expression vectors for held-out test spots of linear models by resolution in µm with cross validation indicated as point shape and line style with relative outperformance of NCEM model versus baseline model. **(d)** Spatial dependencies of individual spot clusters. Shown are R^2 for held-out test data of linear NCEM model by resolution in µm for different spot clusters (n is the number of cells per data set partition as indicated in the each plot title). For each box the centerline defines the median, the height of the box is given by the interquartile range (IQR), the whiskers are given by 1.5 * IQR and outliers are given as points beyond the minimum or maximum whisker.

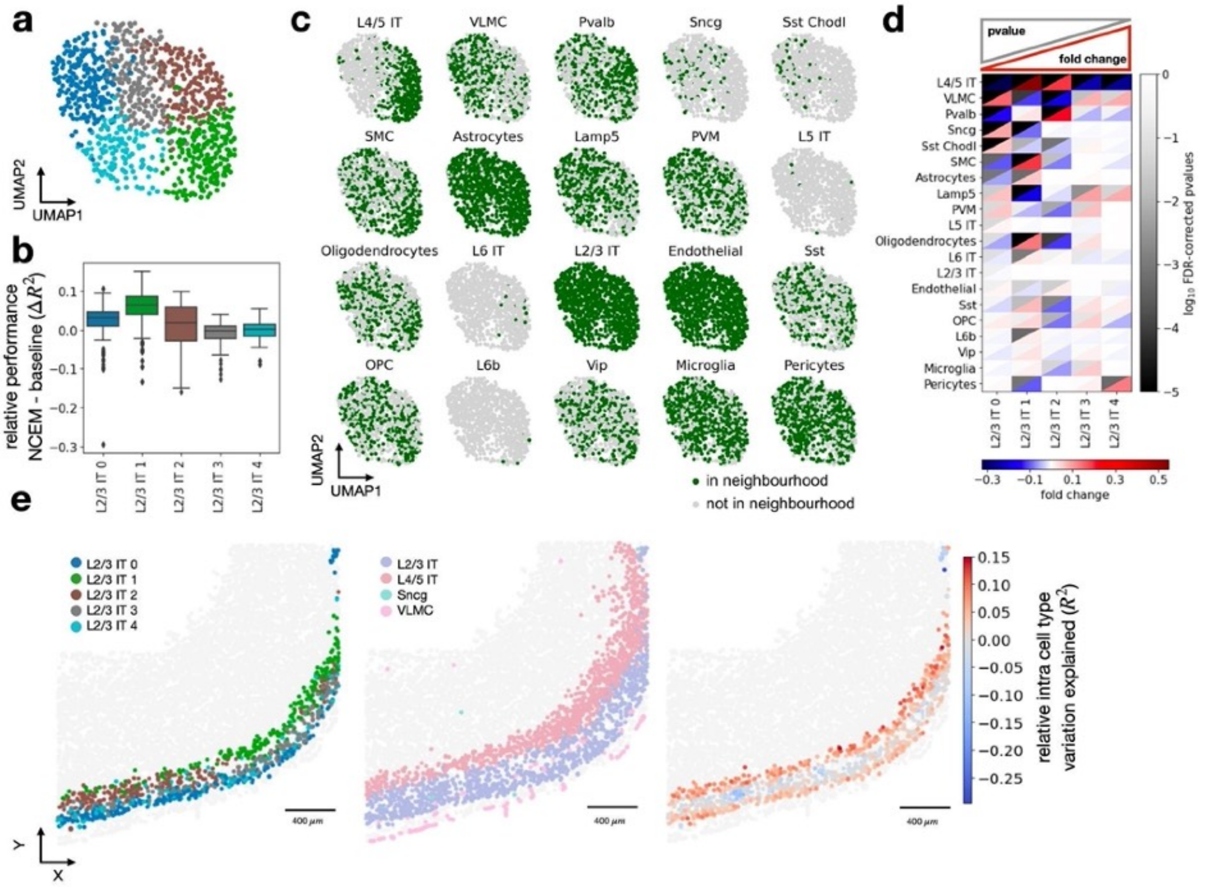

Supp. Fig. 5: Cell heterogeneity can be attributed to niche composition (replicate analysis). (a-e) Replicate analysis corresponding to results presented in Extended Data Fig. 5 on a second image from the MERFISH – brain dataset. **(a)** UMAPs of molecular embedding of L2/3 IT cells only with molecular sub-clustering superimposed (colors as in b)**. (b)** Distribution of cell-wise difference of R^2 between spatial model non non-spatial baseline model by molecular sub-cluster (L2/3 IT 0: n = 226, L2/3 IT 1: n = 209, L2/3 IT 2: n = 193, L2/3 IT 3: n = 191, L2/3 IT 4: n = 127). The centerline of the boxplots defines the median, the height of the box is given by the interquartile range (IQR), the whiskers are given by 1.5 * IQR and outliers are given as points beyond the minimum or maximum whisker. **(c)** UMAPs of molecular embedding of all L2/3 IT cells in example image (n = 946 cells) showing if a given cell-type is present in the neighborhood. The underlying neighborhoods were defined at the optimal resolution identified in Fig. 1d (100 µm). **(d)** Heatmap of fold change and false-discovery rate corrected p-values of cluster enrichment of binary neighborhood labels, where fold changes are the ratio between the relative neighboring source cell-type frequencies per subtype cluster and the overall source cell-type frequency in the image. **(e)** Model performance on L2/3 IT cells in space on slice 162 of mouse brain in the MERFISH – brain with L2/3 IT sub-states (first panel), L2/3 IT, L4/5 IT, Sncg, and VLMC (second panel) and the difference of R2 between the NCEM at resolution of 100 µm and the best nonspatial baseline model (third panel) superimposed.

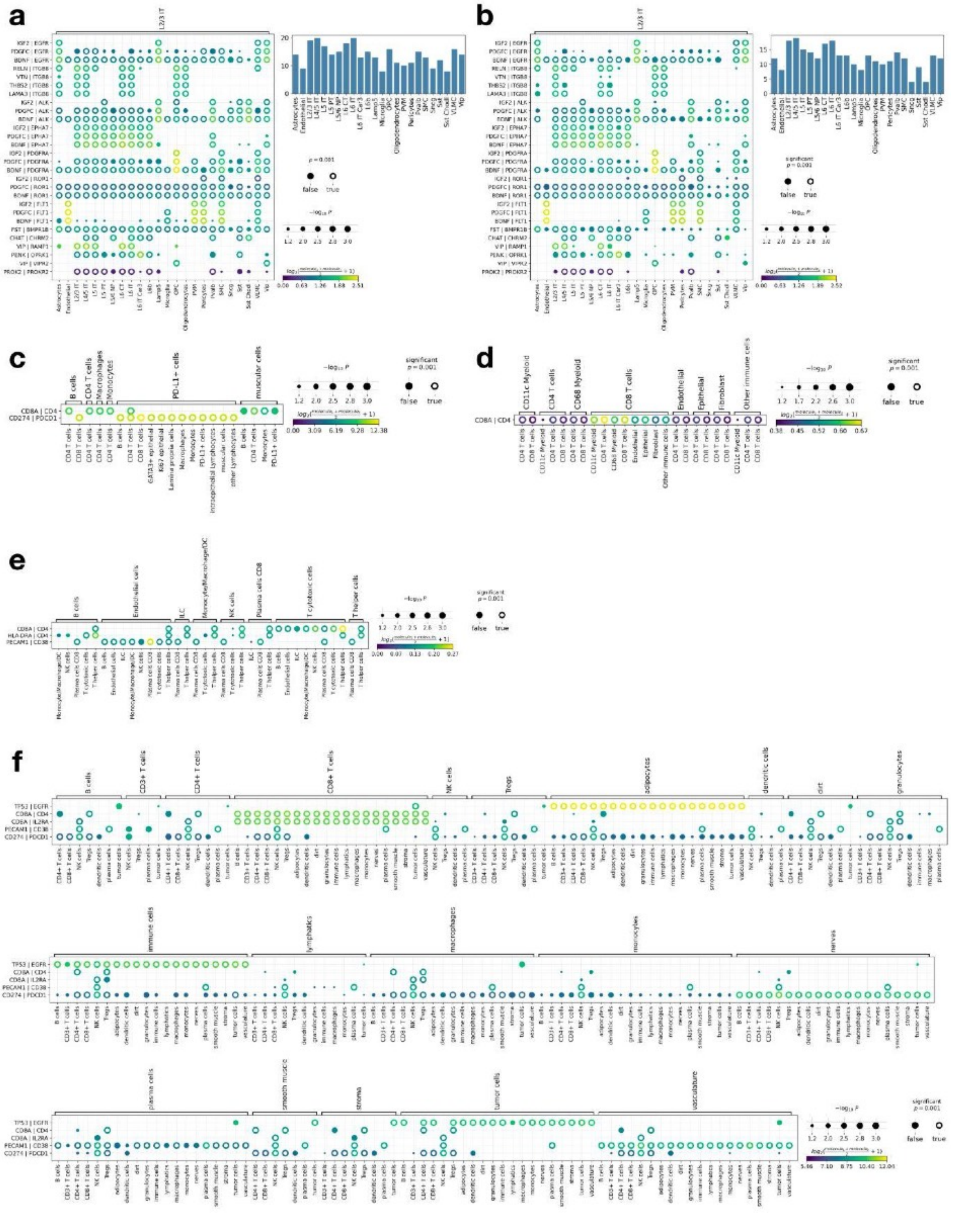

Supp. Fig. 6: Ligand–receptor permutation test. Shown are ligand–receptor gene pairs observed in the study and their significant associations across cell-types for pairs with an empirical p-value from CellPhoneDB analysis ≤ 0.3 for MERFISH – brain (complete dataset L2/3 IT neurons) **(a)**, MERFISH – brain (slice 153 L2/3 IT neurons) **(b)**, chip cytometry – colon **(c)**, MIBI TOF – cancer **(d)**, MELC – tonsils **(e)**, and CODEX – cancer **(f)**. The barplots in **(a, b)** correspond to the count over non-corrected p-values below a threshold of 0.05 per L2/3 IT-source pair.

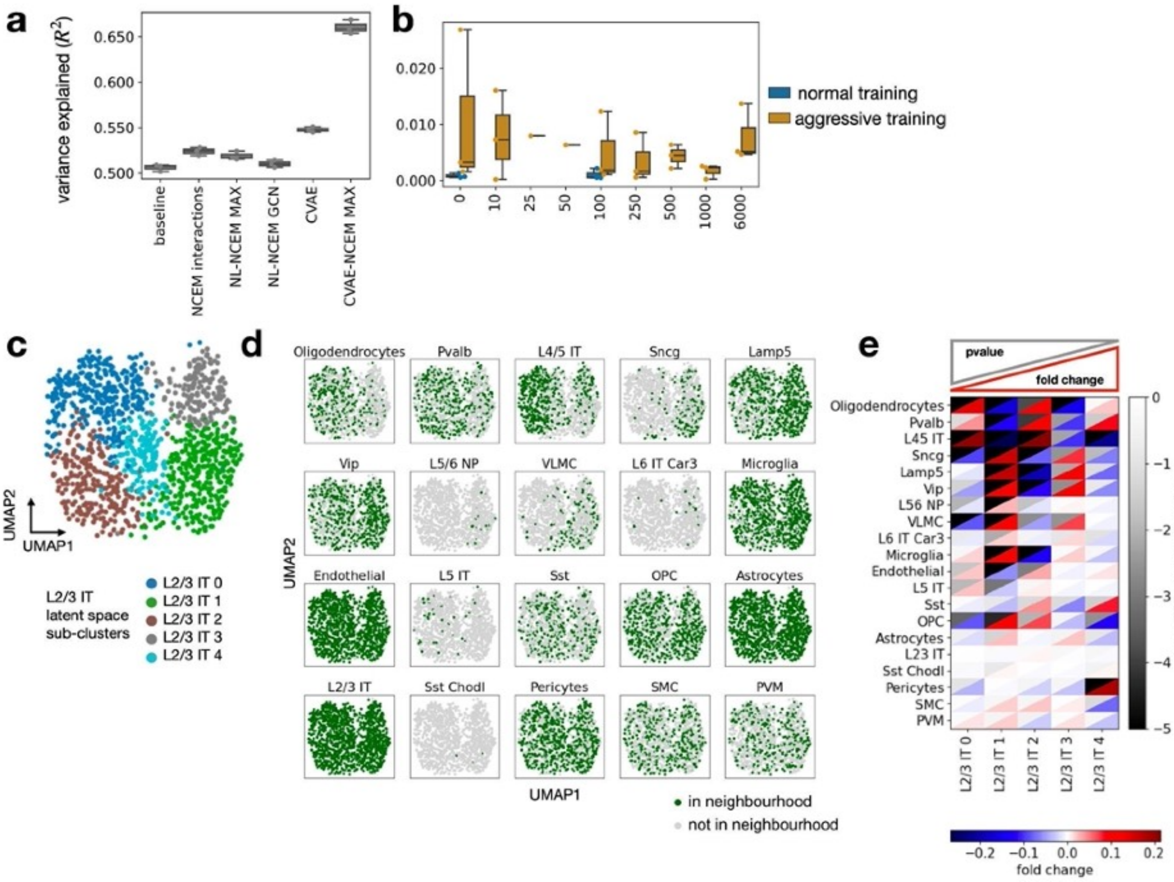

Supp. Fig. 7: CVAE–NCEMs on MERFISH – brain data. (a) Latent variable models improve reconstructive performance. Shown is the R^2 of held-out test data based on forward pass model evaluation from MERFISH – brain data for linear models, nonlinear models and variational autoencoders for both non-spatial and spatial models (n=3 cross-validation splits). *baseline*: a nonspatial linear model of gene expression per cell-type; *NCEM interactions*: linear model with interaction effects; *NL*: nonlinear model; *IND*: the graph kernel is an indicator function across cell-types in the neighborhood; *GCN*: the graph kernel is a graph convolution, a linear embedding of the cell-types in the neighborhood (n=3 rcross-validation splits). **(b)** Neighborhood transfer performance of spatial and non-spatial models. Shown are the R^2 values of cells in the test set for models trained on predicting L2/3 IT cells for both CVAE models CVAE–NCEMs trained on neighborhoods with different radii with optimization algorithm as color. *Plain*: normal CVAE training; *aggressive*: aggressive encoder training. **(c-e)** Latent variables of CVAE–NCEM are confounded with neighborhood conditions. **(c)** UMAP of molecular embedding in the CVAE–NCEM IND latent space of L2/3 IT cells in an example image (n = 1204 cells) with molecular sub-clustering superimposed (L2/3 IT 0: n = 323, L2/3 IT 1: n = 315, L2/3 IT 2: n = 250, L2/3 IT 3: n = 170, L2/3 IT 4: n = 146)**.** **(e)** UMAPs of molecular embedding in the CVAE–NCEM IND latent space of all L2/3 IT cells in the same image with superimposed binary label of presence of a given cell-type, as defined in the title, in the neighborhood. The underlying neighborhoods were defined at a resolution of 100 µm. **(f)** Heatmap of fold change and false-discovery rate corrected p-values of cluster enrichment of binary neighborhood labels, where fold changes are the ratio between the relative neighboring source cell-type frequencies per subtype cluster and the overall source cell-type frequency in the image.

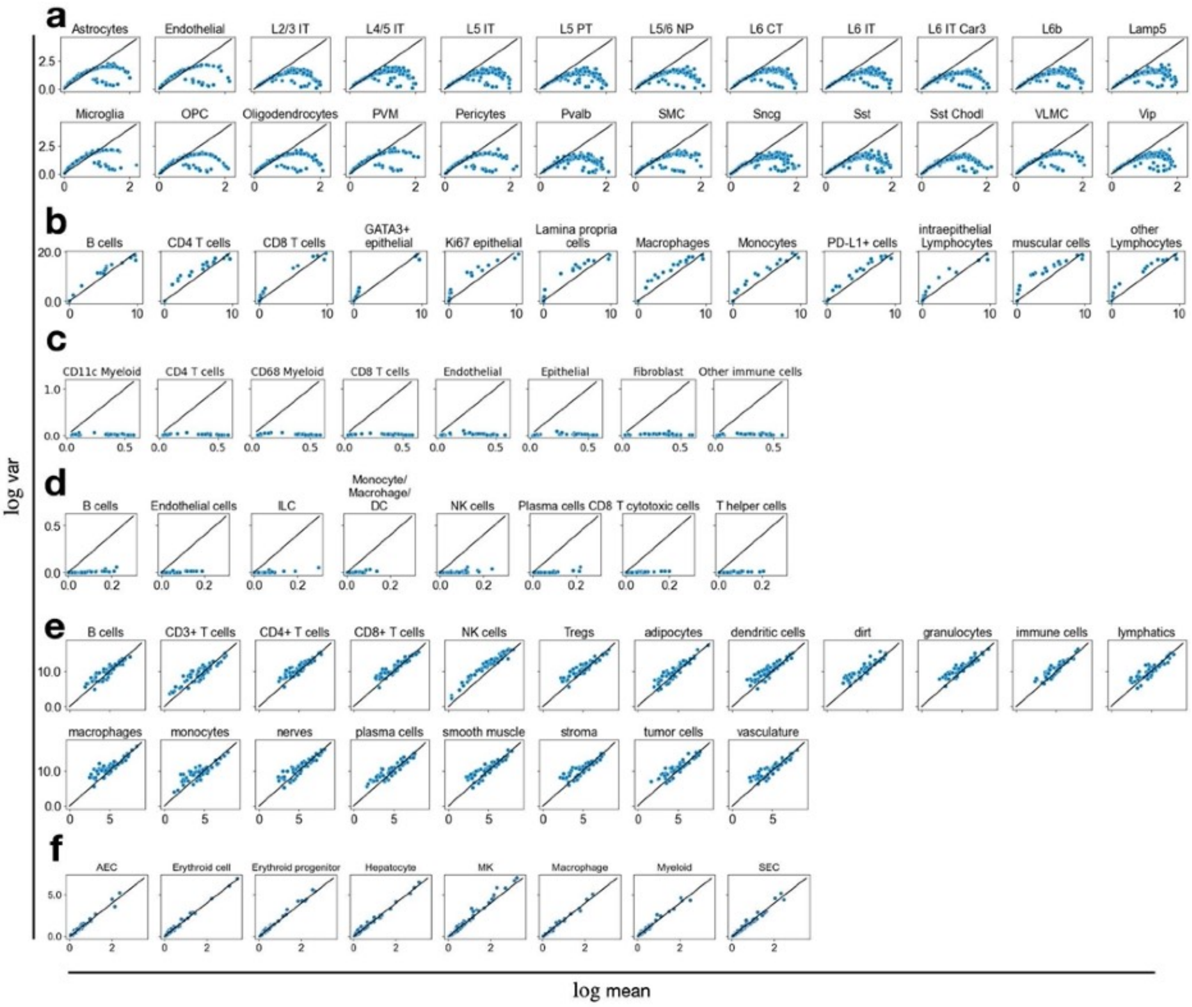

Supp. Fig. 8: Distributional characteristics of gene expression measurements of single cells from spatial molecular profiling assays. Shown is the mean variance plot over observed genes for MERFISH – brain data **(a)**, chip cytometry – colon data **(b)**, MIBI TOF – cancer data **(c)**, MELC – tonsils data **(d)**, CODEX – cancer data **(e)** and MERFISH – wild type fetal liver **(f)**.

method	constrained by niches observed in spatial data	is predictive	models cell-cell communication events in space	works on targeted protocols without capture of cognate ligands and receptors	does not use potentially limited leave-one-gene-out cross-validation
NCEM	yes	yes	yes	yes	yes
SpaOTsc[1]	yes		yes		yes
SVCA[2]	yes	yes	yes	yes	
Garcia-Alonso *et al.*[3]	yes		yes		yes
GNCG[4]	yes				yes
MISTy[5]	yes	yes	yes	yes	
stLearn[6]	yes		yes	yes	
Neighborhood enrichment: Giotto[7] squidpy[8]	yes			yes	yes

Supp. Table 1: Models of dependencies between cells in spatial molecular profiling data. Key algorithm design choices for each method: NCEM, SpaOTsc, SVCA, Garcia-Alonso et al., GNCG, MISTy, stLearn, and neighbourhood enrichment with Giotto and squidpy. We compare if the methods use local spatial constraints, if they are predictive, if they are models of cell communication events, if they are strictly based on ligand-receptor interactions and thus not suitable to targeted assays with few genes captures, and if they are evaluated based on leave-one-gene-out cross-validation. We considered a model to be predictive if it builds a parametric model for spatial dependencies.

method	models source-target interactions	models niche motives of higher order (>2 participating cells)	models intrinsic variation alongside extrinsic effects from niche	feature space for interaction modeling
linear NCEM	yes	no	no	categorical cell types
nonlinear NCEM	yes	yes	no	categorical cell types
nonlinear NCEM-LR	yes	no	no	ligand and receptor gene expression
CVAE-NCEM	yes	yes	yes	categorical cell types

Supp. Table 2: Comparison of model features of NCEM variants.

dataset / first author	technology	domain	transform	node size scaling	batch size	n
**MERFISH – fetal liver (wild type)**	MERFISH	image	log(x+1)	False	140	10
**MERFISH – fetal liver (Tet2$^{-/-}$)**	MERFISH	patient	log(x+1)	False	195	10
**MERFISH – brain**	MERFISH	patient	-	False	64	10
**chip cytometry – colon**	Chip Cytometry	patient	log(x+1)	False	2	100
**MIBI TOF – cancer**	MIBI-TOF	image	-	True	58	10
**MELC – tonsils**	MELC	patient	-	True	1	200
**CODEX – cancer**	CODEX	patient	log(x+1)	True	140	10
**Visium – lymph node**	10x Visium	patient	-	False	1	10

Supp. Table 3: Overview of datasets analyzed in this study. Shown are the spatial molecular profiling chemistry, the domain effect accounted for via batch covariates, the transformation used on the expression vectors, the inclusion of cell size factors, the number of images given to the models during each update (batch size) and the number of nodes evaluated per image per batch (n).

dataset	learning rates	L1/L2
MERFISH — fetal liver	0.05	[0., 1., 0.1, 1e-3]
MERFISH — brain	0.05	0.
Chip Cytometry — colon	0.05	[0., 1., 0.1]
MIBI-TOF — cancer	0.05	[0., 1., 0.1]
MELC — tonsils	0.05	0.
CODEX — cancer	0.05	0.
Visium – lymph node	0.05	0.

Supp. Table 4: Overview of model architecture hyper-parameters tested in grid searches for linear models. Shown are the model hyperparameters screened for the results shown in Fig. 1c. Refer to Supp. Table 3 for hyper-parameters related to data format.

dataset	learning rates	L1	L2	Latent dim.	dropout	hidden layers	Intermediate dim.
MERFISH – fetal liver	0.05	0.	1e-6	[12, 24, 48]	0.05	[0, 1]	[64, 128]
MERFISH – brain	0.05	0.	1e-6	[24, 48]	0.05	[0, 1]	[64, 128]
Chip Cytometry – colon	0.05	0.	1e-6	[12, 24]	0.05	[0, 1]	[16, 32, 64, 128]
MIBI-TOF – cancer	0.05	[0., 1., 0.1]	[0., 1., 0.1]	[8, 16]	0.05	[0, 1]	[4, 8, 32]

Supp. Table 5: Overview of model architecture hyper-parameters tested in grid searches for nonlinear and CVAE models. Shown are the model hyperparameters screened for the results shown in Extended Data Fig 9, Supp. Fig. 7. Refer to Supp. Table 3 for hyper-parameters related to data format.

1. Cang, Z. & Nie, Q. Inferring spatial and signaling relationships between cells from single cell transcriptomic data. Nat. Commun. 11, 2084 (2020).
2. Arnol, D., Schapiro, D., Bodenmiller, B., Saez-Rodriguez, J. & Stegle, O. Modeling Cell-Cell Interactions from Spatial Molecular Data with Spatial Variance Component Analysis. Cell Rep. 29, 202–211.e6 (2019).
3. Garcia-Alonso, L., Handfield, L. F. & Roberts, K. Mapping the temporal and spatial dynamics of the human endometrium in vivo and in vitro. bioRxiv (2021).
4. Yuan, Y. & Bar-Joseph, Z. GCNG: graph convolutional networks for inferring gene interaction from spatial transcriptomics data. Genome Biol. 21, 300 (2020).
5. Tanevski, J., Gabor, A., Flores, R. R., Schapiro, D. & Saez-Rodriguez, J. Explainable multi-view framework for dissecting intercellular signaling from highly multiplexed spatial data. doi:10.21203/rs.3.rs-735362/v1.
6. Pham, D. T. et al. stLearn: integrating spatial location, tissue morphology and gene expression to find cell types, cell-cell interactions and spatial trajectories within undissociated tissues. bioRxiv (2020).
7. Dries, R. et al. Giotto: a toolbox for integrative analysis and visualization of spatial expression data. Genome Biol. 22, 78 (2021).
8. Palla, G. et al. Squidpy: a scalable framework for spatial omics analysis. Nat. Methods 19, 171–178 (2022).
